# Distinct histopathological phenotypes of severe alcoholic hepatitis suggest different mechanisms driving liver injury and failure

**DOI:** 10.1172/JCI157780

**Published:** 2022-07-15

**Authors:** Jing Ma, Adrien Guillot, Zhihong Yang, Bryan Mackowiak, Seonghwan Hwang, Ogyi Park, Brandon J. Peiffer, Ali Reza Ahmadi, Luma Melo, Praveen Kusumanchi, Nazmul Huda, Romil Saxena, Yong He, Yukun Guan, Dechun Feng, Pau Sancho-Bru, Mengwei Zang, Andrew MacGregor Cameron, Ramon Bataller, Frank Tacke, Zhaoli Sun, Suthat Liangpunsakul, Bin Gao

**Affiliations:** 1 Laboratory of Liver Diseases, National Institute on Alcohol Abuse and Alcoholism (NIAAA), NIH, Bethesda, Maryland, USA.; 2 Division of Gastroenterology and Hepatology, Department of Medicine, Indiana University School of Medicine, Indianapolis, Indiana, USA.; 3 Department of Hepatology and Gastroenterology, Charité Universitätsmedizin Berlin, Campus Virchow-Klinikum and Campus Charité Mitte, Berlin, Germany.; 4 Department of Surgery, Johns Hopkins University School of Medicine, Baltimore, Maryland, USA.; 5 Center for Liver Diseases, University of Pittsburgh Medical Center, Pittsburgh, Pennsylvania, USA.; 6 Department of Pathology and Laboratory Medicine, Indiana University School of Medicine, Indianapolis, Indiana, USA.; 7 Department of Pathology and Laboratory Medicine, Emory University School of Medicine, Atlanta, Georgia, USA.; 8 Institut d’Investigacions Biomèdiques August Pi i Sunyer (IDIBAPS), University of Barcelona, Centro de Investigación Biomédica en Red de Enfermedades Hepáticas y Digestivas (CIBERehd), Barcelona, Spain.; 9 Department of Molecular Medicine, Barshop Institute for Longevity and Aging Studies, Center for Healthy Aging, University of Texas Health San Antonio, San Antonio, Texas, USA.; 10 Geriatric Research, Education and Clinical Center, South Texas Veterans Health Care System, San Antonio, Texas, USA.; 11 Department of Biochemistry and Molecular Biology, Indiana University School of Medicine, Indianapolis, Indiana, USA.; 12 Roudebush Veterans Administration Medical Center, Indianapolis, Indiana, USA.

**Keywords:** Gastroenterology, Hepatitis

## Abstract

Intrahepatic neutrophil infiltration has been implicated in severe alcoholic hepatitis (SAH) pathogenesis; however, the mechanism underlying neutrophil-induced injury in SAH remains obscure. This translational study aims to describe the patterns of intrahepatic neutrophil infiltration and its involvement in SAH pathogenesis. Immunohistochemistry analyses of explanted livers identified two SAH phenotypes despite a similar clinical presentation, one with high intrahepatic neutrophils (Neu^hi^), but low levels of CD8^+^ T cells, and vice versa. RNA-Seq analyses demonstrated that neutrophil cytosolic factor 1 (NCF1), a key factor in controlling neutrophilic ROS production, was upregulated and correlated with hepatic inflammation and disease progression. To study specifically the mechanisms related to Neu^hi^ in AH patients and liver injury, we used the mouse model of chronic-plus-binge ethanol feeding and found that myeloid-specific deletion of the *Ncf1* gene abolished ethanol-induced hepatic inflammation and steatosis. RNA-Seq analysis and the data from experimental models revealed that neutrophilic NCF1-dependent ROS promoted alcoholic hepatitis (AH) by inhibiting AMP-activated protein kinase (a key regulator of lipid metabolism) and microRNA-223 (a key antiinflammatory and antifibrotic microRNA). In conclusion, two distinct histopathological phenotypes based on liver immune phenotyping are observed in SAH patients, suggesting a separate mechanism driving liver injury and/or failure in these patients.

## Graphical abstract

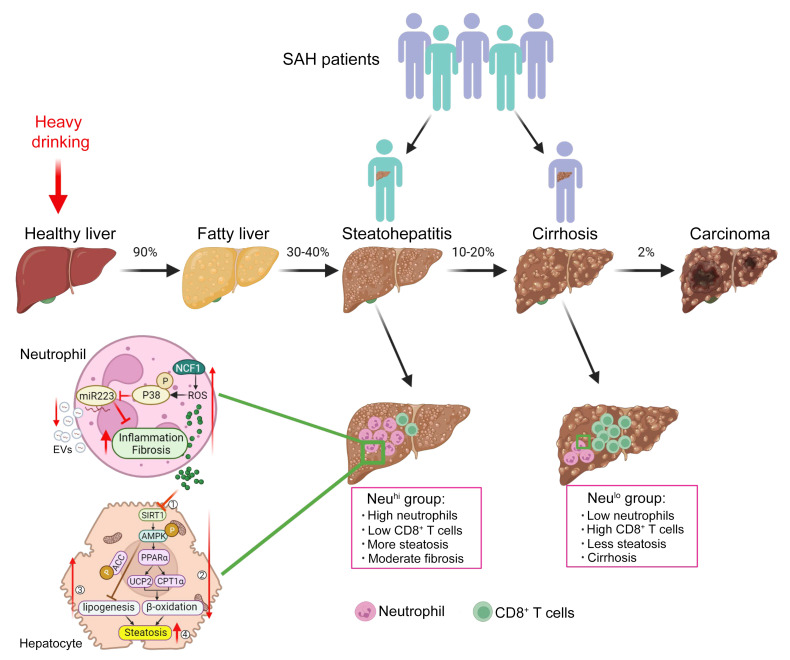

## Introduction

Alcohol use disorder is a major contributor to several medical morbidities, including alcohol-associated liver disease (ALD) (1). There has been an exponential increase in the incidence of ALD worldwide, particularly in the United States (2). Patients with ALD may present with various histopathological abnormalities ranging from simple steatosis to more severe manifestations associated with inflammation and fibrosis (3–5). Alcoholic hepatitis (AH) is characterized by an abrupt rise in serum bilirubin levels, systemic inflammation, coagulopathy, and liver-related complications. It has a high short-term mortality, especially in patients with severe forms of AH (SAH) (6, 7). Despite the advancement in basic and translational research activities, the underlying molecular mechanisms driving AH pathogenesis remain poorly understood (8, 9). At present, no FDA-approved pharmacological interventions have been approved (3–5, 10, 11). Accumulating evidence suggests that multiple types of immune cells are involved in the pathogenesis of AH, including neutrophils, resident and infiltrating macrophages, and other cell types in the innate and adaptive immune system (12). The ratio of neutrophils to lymphocytes has been reported to predict 90-day mortality of AH patients (13). However, the role and function of immune cells in the pathogenesis of AH remain obscure.

Neutrophil dysfunction has been described not only in patients with AH, but also in those with cirrhosis (14, 15). However, the mechanisms underlying neutrophil dysfunction in ALD and its implications in alcohol-induced liver injury are not well understood. Neutrophils are the most abundant white blood cells in mammals, representing the first line of innate immune response. Neutrophils in the peripheral blood were previously thought to be a homogeneous cell type. However, recent data have revealed the heterogeneity of neutrophil populations with varying functions beyond antimicrobial properties. Neutrophils play an important role in tissue repair and immune regulation (16–18) and thus likely play multiple roles in alcohol-induced liver injury by coordinating the immune and inflammatory responses through phagocytosis, ROS generation, degranulation, cytokine and chemokine production, and neutrophil extracellular trap (NETs) release (19–22). Neutrophil-induced oxidative stress and inflammation can be triggered by the oxidative modification of albumin found in patients with SAH (20). We have previously reported that neutrophils contribute to alcohol-induced liver injury, as demonstrated by findings that blocking neutrophil infiltration using anti–C-X-C motif chemokine ligand 1 (anti-CXCL1) treatment or *Cxcl1* gene deletion significantly ameliorated alcohol-induced liver damage (19).

Mechanistically, neutrophils cause tissue injury by producing inflammatory mediators, proteases, and ROS (18). ROS production in neutrophils is controlled by the multimeric transmembrane enzyme complex NADPH oxidase (NOX) (23). In addition, several NOX-associated molecules are required for NOX activation, acting by stabilizing the NOX proteins and docking cytosolic factors, including phagocytic oxidase (phox) units: p47^phox^ (also called neutrophil cytosolic factor 1 [NCF1]), p67phox (NCF-2), p40phox (NCF-4), p22phox, and gp91phox (23). Among them, the *Ncf1* gene, which encodes the phox unit p47phox, is predominantly expressed in neutrophils and plays an important role in controlling ROS production in neutrophils (24).

In the current translational study, we observed an interpatient variability with distinct hepatic inflammatory cell infiltration in patients with SAH. Two interesting observations were (a) a negative correlation between neutrophilic infiltration in the hepatic parenchymal area and CD8^+^ T cells in the fibrotic area and (b) a positive correlation among neutrophilic infiltration, model for end-stage liver disease score (MELD score; a scoring system predicting prognosis of liver disease), and serum alanine aminotransferase (ALT) levels. Mechanistically, we found that hepatic *NCF1* expression is markedly upregulated and correlates with neutrophil infiltration in SAH patients. To define the role of neutrophil NCF1 in AH pathogenesis, we generated myeloid cell–specific *Ncf1*-KO (*Ncf1^Lyz–/–^*) mice and challenged these mice with chronic-plus-binge ethanol feeding, a model known to be associated with high intrahepatic neutrophil infiltration (25). Our data revealed that *Ncf1^Lyz–/–^* mice had reduced hepatic neutrophil accumulation, liver injury, and inflammation compared with ethanol-fed WT mice. Transcriptomic analysis with RNA-Seq demonstrated a unique role of neutrophilic NCF1-induced ROS generation in mediating alcohol-induced liver injury through its regulation of inflammation, lipid metabolism, and fibrosis-related pathways. We observed a significant reduction of AMPK activity and microRNA-223 (miR-223) by NCF1-dependent ROS generation in ethanol-fed mice. AMPK is a central regulator of lipid metabolism (26, 27), while miR-223 plays a key role in limiting liver inflammation, fibrosis, and injury (28–30). Our data provide a mechanistic insight into the role of neutrophils in ALD, suggesting that neutrophilic NCF1-dependent ROS generation promotes steatosis and hepatic inflammation by inhibiting AMPK and miR-223, respectively.

## Results

### Immunohistochemical analyses of inflammatory cells in patients with SAH.

To characterize inflammatory cells in patients with SAH, we performed IHC analyses in 7 SAH explant liver samples. As illustrated in Supplemental Figure 1A (supplemental material available online with this article; https://doi.org/10.1172/JCI157780DS1), there was significant intrahepatic infiltration of macrophages, neutrophils, and T and B cells in the parenchymal and fibrotic regions of SAH patients. To be specific, a large number of CD68^+^ macrophages/Kupffer cells were detected in healthy control livers and in the hepatic parenchyma of SAH patients. A higher number of CD68^+^ macrophages were also observed in the fibrotic area of SAH patients. CD20^+^ B cells were detected in the fibrotic area, with a few in the parenchymal area of SAH patients and healthy controls.

**Figure 1:**
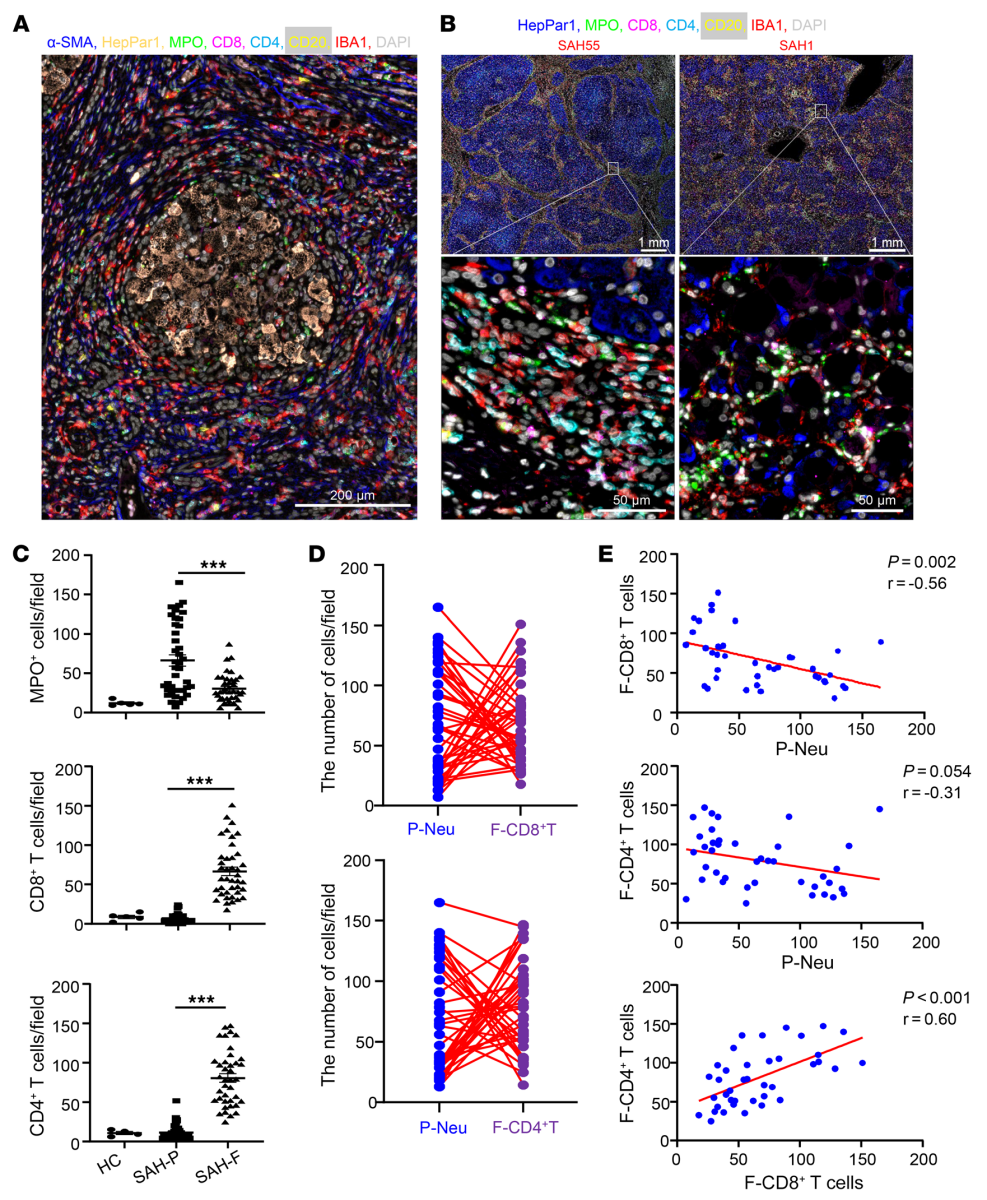
IHC staining analyses of inflammatory cells in the liver of SAH patients. (**A** and **B**) Liver tissues from SAH patients (*n* = 10) were subjected to sequential multiplex immunofluorescence staining of α-SMA, HepPar1, MPO, CD8, CD4, CD20, and IBA1. (**A**) Typical starry image of immunofluorescent staining from 1 SAH patient with a large number of inflammatory cells surrounding hepatocytes. (**B**) Two different patterns of representative images from SAH55 and SAH1 patients. (**C**) Liver tissues from healthy controls (HC) (*n* = 5) and SAH patients (*n* = 40) were subjected to IHC staining for MPO, CD8, and CD4. The number of MPO^+^, CD8^+^, CD4^+^ cells in the parenchymal area (SAH-P) and fibrotic area (SAH-F) were quantified. From 5 to 7 parenchymal or fibrotic areas were randomly selected for quantitation, and average numbers of cells are shown. For the graph, each dot represents one patient. (**D** and **E**) Analyses of SAH data from **C** show negative correlation between P-Neu and F-CD8^+^ T cells or F-CD4^+^ T cells and positive correlation between F-CD8^+^ T cells and F-CD4^+^ T cells. *P* values are indicated. Data are represented as mean ± SEM. ***P < 0.001. Statistical significance was assessed using 1-way ANOVA followed by Tukey’s post hoc test for multiple groups (**C**) and Pearson’s correlation analysis (**E**).

We next conducted sequential multiplex immunostaining with multiple antibodies, including HepPar1 (hepatocyte-specific antigen), myeloperoxidase (MPO), CD8, CD4, CD20, and ionized calcium binding adaptor molecule 1 (IBA1) (a marker for macrophages). A large number of multiple types of inflammatory cells were detected in SAH with 1 representative starry image similar to galaxy that is shown in Figure 1A. Furthermore, we noticed that a significant number of neutrophils were observed in parenchymal areas of SAH patients (Figure 1B), while a large number of CD4^+^ and CD8^+^ T cells were found in fibrotic areas of SAH patients (Figure 1B); this was also supported by neutrophil and T cell density map analysis, respectively (Supplemental Figure 1B).

Interestingly, we observed interpatient variability in the pattern of neutrophil and T cell infiltration. For example, patient SAH55 had much more CD8^+^ T cell staining than MPO^+^ cell staining, while patient SAH1 had much more MPO^+^ cell staining than CD8^+^ T cell staining in the liver (Supplemental Figure 1C). To further characterize this observation, we performed IHC analyses in an additional 33 SAH samples (a total of 40 SAH cases), which were quantified and summarized in Figure 1C. Among SAH patients, the number of MPO^+^ neutrophils was much higher in the parenchymal regions than in the fibrotic areas, while CD4^+^ and CD8*^+^* T cells were mainly detected in the fibrotic areas (Figure 1C). A negative correlation of neutrophils in parenchymal areas (P-neutrophils [P-Neu]) with CD8^+^ T cells in fibrotic areas (F-CD8^+^ T cells) is summarized in Figure 1, D and E. We also observed a negative trend in the number of P-neutrophils and CD4^+^ T cells in fibrotic areas (F-CD4^+^ T cells) (Figure 1E). Intriguingly, a positive correlation was observed between F-CD8^+^ T cells and F-CD4^+^ T cells (Figure 1E).

### Correlation of hepatic neutrophil and CD8^+^ T cell infiltration with MELD score and serum transaminases in SAH patients.

**Figure 2:**
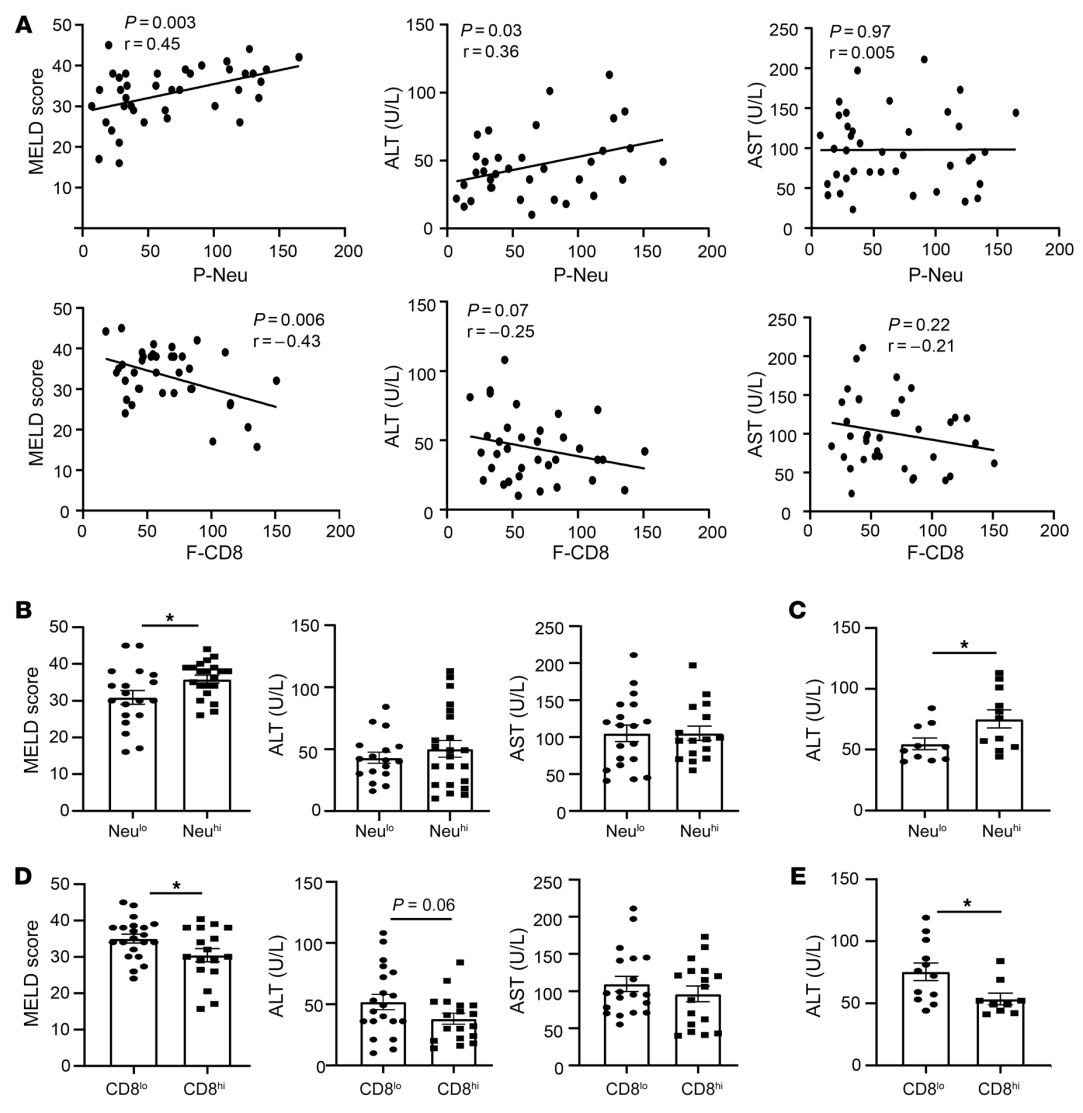
Hepatic neutrophil and CD8^+^ T cell infiltration are associated with liver injury in patients with SAH. (**A**) Upper panel: correlation analysis between the numbers of P-Neu per field and MELD score, serum ALT, or AST level; lower panel: correlation analysis between the numbers of F-CD8^+^ T cells per field and MELD score, serum ALT, or ALT level. (**B**–**E**) SAH patients were dichotomized into Neu^lo^ and Neu^hi^ groups (using the average quantity at 50 cells/field of intrahepatic neutrophils in our study cohort as the cutoff) or into CD8^lo^ and CD8^hi^ groups. MELD score, serum AST, and ALT levels were compared between groups. For panels **B** and **D**, all SAH patients were used for the comparison. For panels **C** and **E**, only SAH patients with abnormal ALT (>40 U/L) levels were used for the comparison. Data are represented as mean ± SEM. *P < 0.05. Statistical significance was assessed using Pearson’s correlation analysis (**A**) and 2-tailed Student’s *t* test for comparing 2 groups (**B**–**E**).

To determine whether hepatic neutrophil and CD8^+^ T cell infiltration are associated with liver injury, we analyzed their correlation with MELD scores and serum aminotransaminase levels. As illustrated in Figure 2A, a correlation analysis revealed that hepatic P-Neu infiltration positively correlated with MELD score and serum ALT levels, but not with aspartate aminotransferase (AST) levels, while hepatic F-CD8^+^ T cell infiltration negatively correlated with MELD score and a negative correlation tendency (*P* = 0.07) with ALT levels. We also found a trend in negative correlation between F-CD4^+^ T cells and MELD scores (Supplemental Figure 2A), while we did not find the association among age, MELD score, and ALT levels (Supplemental Figure 2B). To further examine the roles of neutrophils and CD8^+^ T cells in SAH pathogenesis, we divided the patients into 2 groups using the average neutrophils as a cutoff at 50 cells/field. Our data revealed that patients with high P-neu (Neu^hi^) had higher MELD scores than those with low P-neu (Neu^lo^), while serum ALT and AST levels were comparable (Figure 2B). When selecting the subset of patients with ALT elevation (using 40 U/L as a cutoff based on the upper limit of normal ALT levels in the lab, 7–35 U/L in women and 7–40 U/L in men), we found that patients with high P-neu had higher ALT levels than those with low P-neu (Figure 2C). In contrast, patients with higher numbers of CD8^+^ T cells had lower MELD scores than those with lower numbers of CD8^+^ cells (Figure 2D). There was a trend toward lower serum ALT levels in patients with higher numbers of CD8^+^ T cells (*P* = 0.06; Figure 2D). The difference was statistically significant when the analyses were limited to patients who had elevated ALT (above 40 U/L) (Figure 2E).

Patients with high P-neu were younger than those with low P-neu, while patients with higher numbers of F-CD8^+^ cells were older than those with lower numbers of F-CD8^+^ T cells (Supplemental Figure 2C). However, there was no correlation between age and the number of neutrophils or CD8^+^ T cells (Supplemental Figure 2D). We observed a positive correlation between P-neu and circulating peripheral blood neutrophil counts (Supplemental Figure 2E). Additionally, we also found a positive association between peripheral blood neutrophil counts and serum AST and a positive trend between serum ALT levels and MELD scores (Supplemental Figure 2F).

### RNA-Seq analyses focusing on inflammatory cell–related signature genes in patients with SAH.

**Figure 3:**
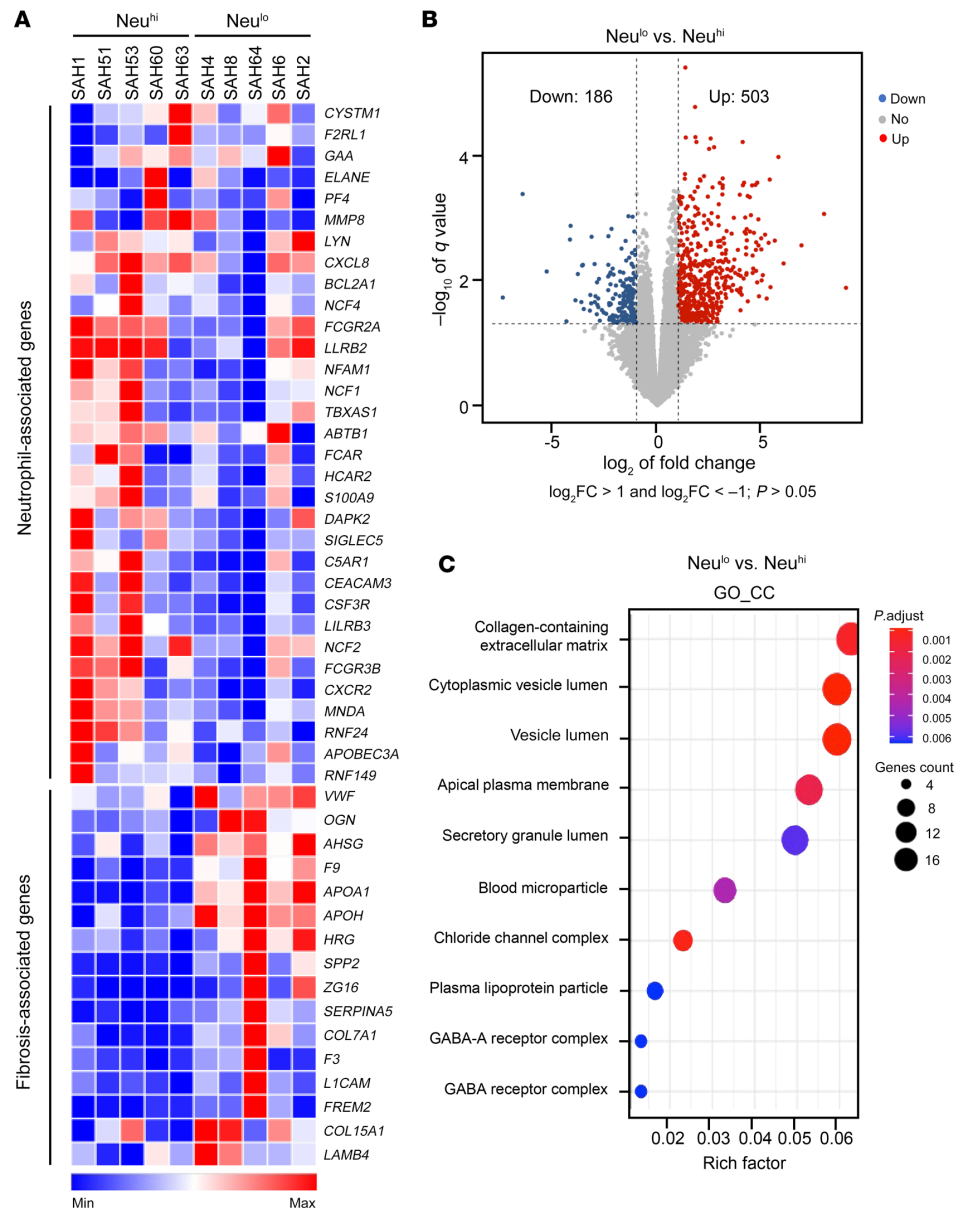
RNA-Seq analyses of the liver tissues of SAH patients (*n* = 10) with Neu^lo^ or Neu^hi^. (**A**) Heatmap analysis of neutrophil- and fibrosis-associated genes in GO terms of the collagen-containing extracellular matrix. (**B**) Volcano plot displaying DEGs comparing Neu^lo^ to Neu^hi^ group. Significant DEGs were at threshold, with less than –1 log_2_ fold change (log_2_FC) greater than 1 and with *P* > 0.05. Dots in red represent upregulated genes (503 genes), while dots in blue represent downregulated genes (186 genes) in Neu^lo^ versus Neu^hi^ samples. (**C**) Upregulated genes (503 genes) in Neu^lo^ samples were subjected to pathway enrichment analysis. Dot plot was used to present the top 10 enriched GO terms in cellular components (GO_CC). Size of the dots represents the number of genes, and color represents *P*-adjusted (*P*.adjust) values.

We employed RNA-Seq to gain mechanistic insight into the infiltration of inflammatory cells in the liver of patients with SAH (3 women, 7 men). Heatmap analysis revealed a significant increase in the hepatic expression of neutrophil and CD8^+^ T cell–related signature genes in SAH patients when compared with healthy controls (Supplemental Figure 3, A and B). The higher expression of neutrophil-related signature genes corresponded to the higher neutrophil infiltration based on IHC staining, notably for SAH patients (SAH1, SAH51, SAH53, SAH60, and SAH63; Figure 3A). An increase in the expression of CD8^+^ T cell–related signature genes was observed in SAH patients SAH2, SAH4, SAH8, and SAH64 (Supplemental Figure 3C). We next analyzed differentially expressed genes (DEGs) in SAH patients stratified by the quantity of hepatic neutrophils and Neu^lo^ and Neu^hi^ groups based on the IHC findings, as outlined above. The volcano plot illustrated 503 up- and 186 downregulated hepatic genes in SAH patients when we compared the gene expression in patients with Neu^lo^ and those with Neu^hi^ (Figure 3B). We next performed a pathway enrichment analysis of the 503 upregulated genes; among the top 10 enriched Gene Ontology (GO) terms in cellular components (GO_CC), genes associated with the collagen-containing extracellular matrix had the highest expression (Figure 3C).

### Association between fibrosis scores and inflammatory cell infiltration in patients with SAH and alcoholic steatohepatitis.

**Figure 4:**
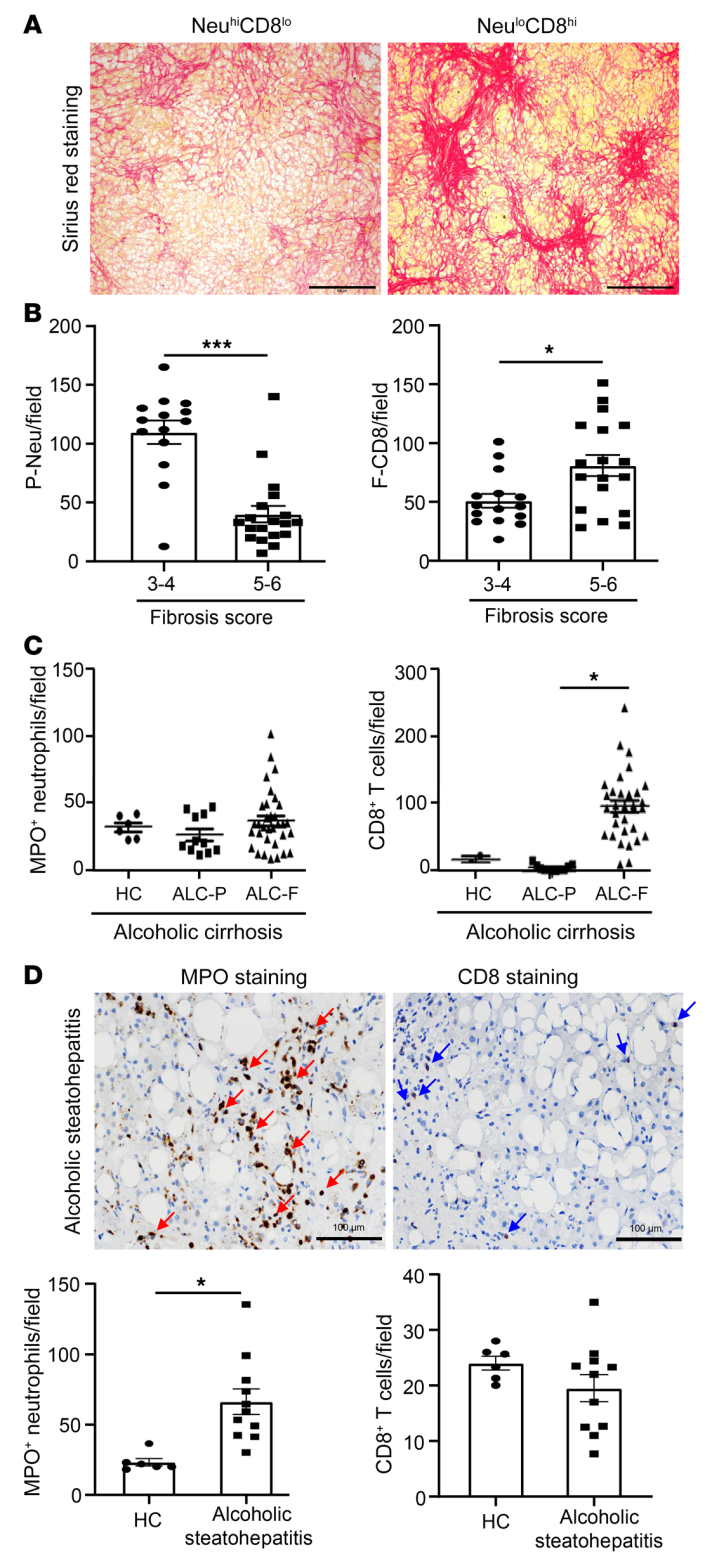
Hepatic neutrophil infiltration negatively correlates with fibrosis progression in patients with SAH, cirrhosis, and alcoholic steatohepatitis. (**A** and **B**) Liver tissues from SAH patients (*n* = 33) were subjected to Sirius red staining. Representative images from Neu^hi^CD8^lo^ and Neu^lo^CD8^hi^ groups are shown (**A**). Scale bars: 500 μm. (**B**) Fibrosis scores were evaluated by the Ishak system, and SAH patients were divided into 2 groups based on fibrosis scores and hepatic neutrophils. CD8^+^ T cell numbers were compared between the 2 groups. (**C**) Liver tissues from healthy control (*n* = 6) and alcoholic cirrhosis (ALC) patients (*n* = 32) were subjected to IHC staining of MPO, CD8, and MPO^+^; CD8^+^ cells in the parenchymal area (ALC-P) and fibrotic area (ALC-F) were quantified. (**D**) Liver tissues from healthy controls (*n* = 6) and alcoholic steatohepatitis patients (*n* = 12) were subjected to IHC staining of MPO^+^ and CD8^+^ cells. Representative images (top) and quantification are shown (bottom). Red arrows indicate MPO^+^ staining, and blue arrows indicate CD8^+^ staining. Scale bars: 100 μm. Data are represented as mean ± SEM. *P < 0.05; ***P < 0.001. Statistical significance was assessed using 2-tailed Student’s *t* test for comparing 2 groups (**B** and **D**) and 1-way ANOVA followed by Tukey’s post hoc test for multiple groups (**C**).

A significant increase in the expression of genes related to collagen-containing extracellular matrix in patients with Neu^lo^ led us to carefully examine the presence of fibrosis with Sirius red staining (*n* = 32, SAH patients) and assess its degree and severity using the Ishak score (a scoring system for assessment of fibrosis and necroinflammation). The representative Sirius red staining images demonstrated that patients with Neu^lo^ but CD8^hi^ infiltration had more fibrosis than those with Neu^hi^ but CD8^lo^ infiltration (Figure 4A). The cumulative data of these 32 SAH patients on the quantity of P-neu, F-CD8^+^ T cells, and fibrosis scores are summarized in Figure 4B. SAH patients with a higher fibrosis score or Ishak score of 5 or 6 had a smaller amount of parenchymal neutrophil infiltration, but a high number of CD8^+^ T cells in the fibrosis area when compared with those with a fibrosis score of 3 or 4 (Figure 4B). Additionally, we did not observe a difference in the quantity of CD4^+^ T cell infiltration between patients with fibrosis 3–4 and 5–6 (Supplemental Figure 3D). Based on the histological finding, it is difficult to discern any specific patterns/structures or the zonation in the liver of SAH patients; the findings are varied with inter- and intrapatient heterogeneity. The Sirius red staining (fibrosis areas) was evenly distributed in most SAH patients we stained. Only a small number of SAH patients with moderate fibrosis had both neutrophils and CD8^+^ and CD4^+^ T cells, which was reflected by a comparable number of neutrophils and T cells detected in the same patient, as shown in Figure 1D.

To extend our observation on the hepatic neutrophil and CD8^+^ T cell infiltration in association with fibrosis, we carried out the experiments to include explanted livers from patients with alcoholic cirrhosis and liver biopsy samples from patients with alcoholic steatohepatitis, covering the whole spectrum of ALD clinical presentation. As illustrated in Figure 4C and Supplemental Figure 4, A and B, for patients with cirrhosis, IHC analyses illustrated significant intrahepatic infiltration of CD3^+^ T cells, Foxp3^+^ Tregs, IL-17^+^ T cells, CD20^+^ B cells, and CD68^+^ macrophages in the fibrotic areas. Similarly to our observation in SAH patients, the quantity of CD8^+^ T cells was increased in the fibrotic areas compared with the parenchymal areas with no fibrosis. There was no difference in the quantity of intrahepatic neutrophil infiltration in the fibrotic and parenchymal areas.

For the analysis in patients with alcoholic steatohepatitis, we first performed Sirius red staining and confirmed the presence of mild to moderate fibrosis (Supplemental Figure 5A). Immunofluorescence staining analysis revealed the presence of MPO^+^ neutrophils that were enriched in the parenchymal areas accompanied by few CD8^+^ T cells in both parenchymal and fibrotic areas (Figure 4D and Supplemental Figure 5B).

### The expression of hepatic NCF1/p47^phox^ is increased and correlated with the quantity of hepatic neutrophil infiltration in patients with SAH.

**Figure 5:**
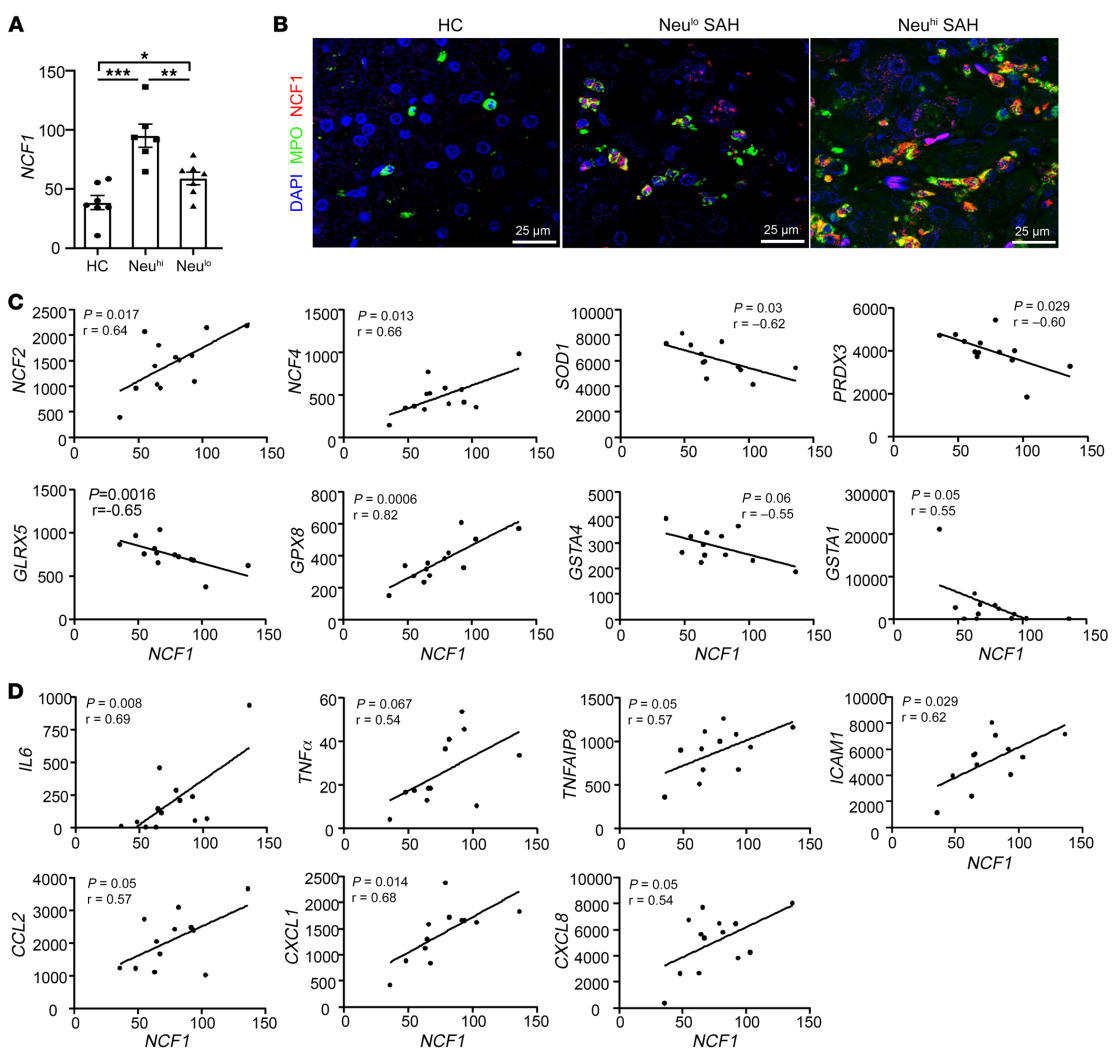
Hepatic levels of *p47phox/NCF1* are elevated and correlated with the quantity of intrahepatic neutrophils in SAH patients. (**A**) Hepatic *NCF1* levels were compared among healthy control (*n* = 7) and Neu^lo^ (*n* = 5) and Neu^hi^ (7) groups, based on RNA-Seq data. (**B**) Liver tissues from SAH patients were subjected to immunofluorescence staining of MPO and NCF1. Representative images are shown. Scale bars: 25 μm. (**C** and **D**) Correlation analyses were performed between *NCF1* and inflammation-related genes or oxidative stress–related genes based on RNA-Seq data (*n* = 13). Values in the *x* and *y* axes represent relative fragments per kilobase of exon per million mapped fragments (FPKM) from RNA-Seq data. Data in **A** are represented as mean ± SEM. *P < 0.05; **P < 0.01; ***P < 0.001. Statistical significance was assessed using 1-way ANOVA followed by Tukey’s post hoc test for multiple groups (**A**) and Pearson’s correlation analysis (**C** and **D**).

As previously mentioned, neutrophils cause tissue injury by producing inflammatory mediators, proteases, and ROS. Among all neutrophil-related signature genes, the *NCF1* gene, encoding for the cytosolic 47 kDa phagocytic NADPH oxidase (p47phox) (31), plays an important role in controlling ROS production in neutrophils. Our RNA-Seq and heatmap analysis shown in Supplemental Figure 3A demonstrated a significant increase in the expression of *NCF1* among patients with SAH; its expression was significantly higher in SAH patients with Neu^hi^ when compared with those in the Neu^lo^ groups, which is also summarized in Figure 5A. We next performed immunofluorescence analysis by costaining the neutrophils with MPO and NCF1 and again found an increase in the expression of NCF1 in parallel with the quantity of intrahepatic neutrophils (Figure 5B). We also performed immunoblot analysis for hepatic NCF1 and found an increase in hepatic NCF1 expression in SAH patients when compared with healthy controls (Supplemental Figure 6A). Among SAH samples, there was an increasing trend in hepatic NCF1 expression in Neu^hi^ patients compared with Neu^lo^ patients (Supplemental Figure 6A). To test the hypothesis that an induction of NCF1 in SAH patients led to an increase in ROS production, we performed IHC analysis by staining for malondialdehyde (MDA) and 4-hydroxynonenal (4-HNE), the byproducts of lipid peroxidation. We found a significant increase in hepatic MDA and 4-HNE in patients with SAH (Supplemental Figure 6B). We also observed that hepatic *NCF1* expression positively correlated with several oxidative stress–related genes, such as *NCF2*, *NCF4*, and glutathione peroxidase 8 (*GPX8*). Of interest, *NCF1* expression was negatively correlated with superoxide dismutase 1 (*SOD1*), peroxiredoxin 3 (*PRDX3*), and glutaredoxin 5 (*GLRX5*) (Figure 5C).

In addition to ROS production, neutrophils cause tissue injury through the production of inflammatory mediators. We next performed correlation analyses between the expression of hepatic *NCF1* and genes associated with inflammatory pathways in patients with SAH. A positive correlation among *NCF1*, intercellular adhesion molecule 1 ( *ICAM1*), and *CXCL1* was observed with the trend in its correlation with *IL6*, *TNFα*, *TNFAIP8*, *CCL2*, and *CXCL8* in patients with SAH (Figure 5D); this was reported to be associated with prognosis among AH patients (32, 33). The expression of hepatic *NCF1* was negatively correlated with several antioxidative stress genes, such as *SOD1*, glutathione synthetase (*GSS*), and peroxiredoxin 4 (*PRDX4*) (Supplemental Figure 6, C and D).

### An increase in hepatic NCF1 expression and neutrophil-related genes in patients with alcoholic steatohepatitis.

**Figure 6:**
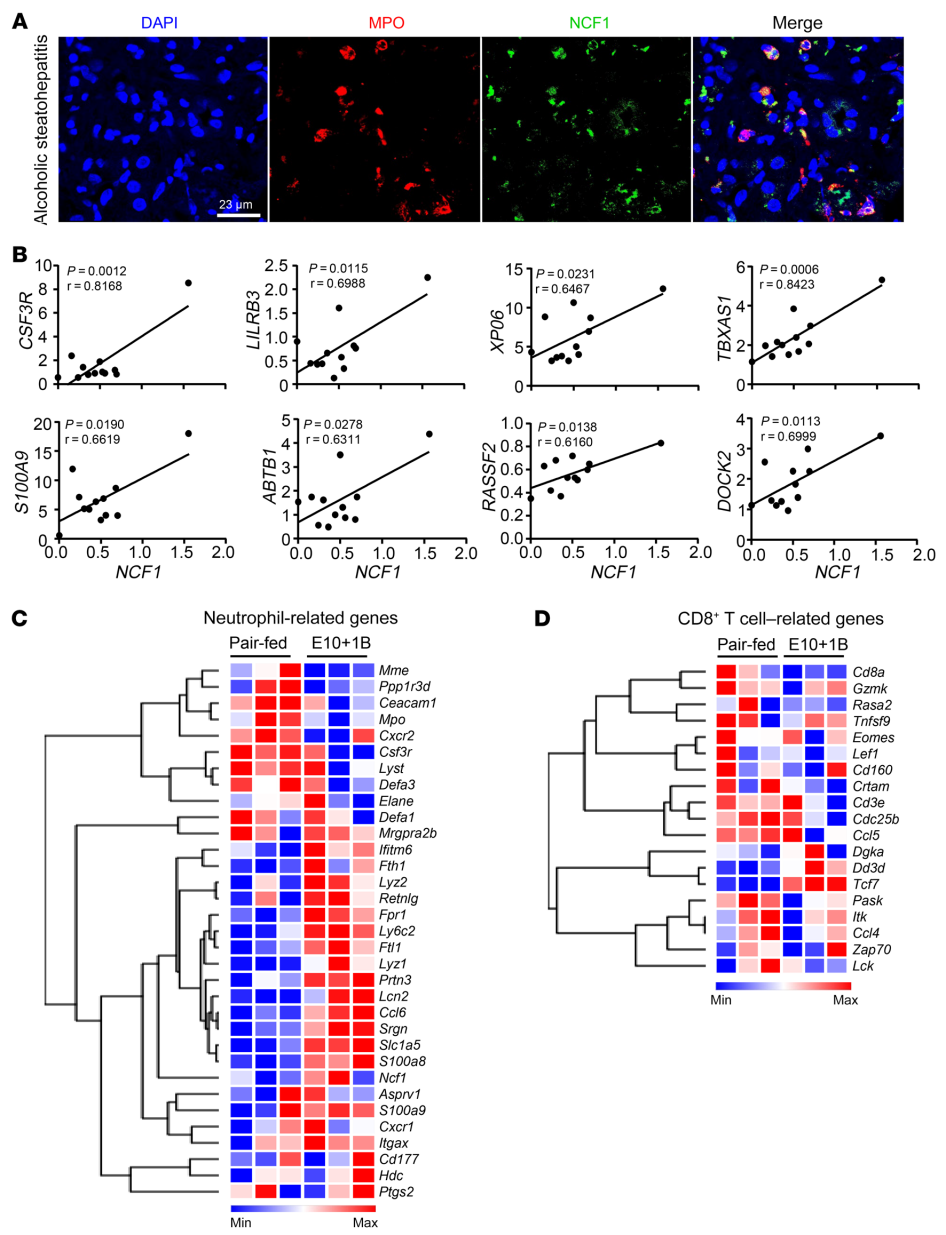
Hepatic NCF1 expression is upregulated and positively correlated with neutrophil-related genes in patients and mice with alcoholic steatohepatitis. (**A**) Liver tissues from alcoholic steatohepatitis patients were subjected to immunofluorescence staining of MPO and NCF1. Representative images are shown. Scale bar: 23 μm. (**B**) Correlation analyses were performed between NCF1 and neutrophil-related genes based on RNA-Seq data (*n* = 12). Values in *x* and *y* axes represent relative transcripts per million (TPM). (**C** and **D**) Liver tissues from ethanol- (*n* = 3) and pair-fed (*n* = 3) mice were subjected to microarray analyses. Heatmap analyses of neutrophil-related genes (**C**) and CD8^+^ T cell–related genes (**D**). Microarray data were pulled from our previous paper (50); GEO database number is GSE67546. Statistical significance was assessed using Pearson’s correlation analysis (**B**).

The above data illustrated an increase in hepatic *NCF1* expression and its correlation with inflammatory genes in SAH patients with liver failure. We next asked whether the expression of hepatic NCF1 was also increased in patients with alcoholic steatohepatitis; this was determined by performing immunofluorescence staining for the presence of inflammatory cells. As revealed in Figure 6A, the expression of NCF1 was elevated in hepatic neutrophils of these patients. Additionally, the correlation analysis between *NCF1* and neutrophil-related gene expression was observed in alcoholic steatohepatitis patients (Figure 6B), which was similar to that of SAH patients with liver failure, as shown in Figure 5.

### Genetic deletion of the Ncf1 gene in neutrophils ameliorates chronic-plus-binge ethanol-induced liver ROS, inflammation, and steatosis.

The above data demonstrate the level of intrahepatic neutrophil infiltration, especially in those with Neu^hi^, in association with liver inflammation and high MELD score. To specifically study the mechanism of how high levels of intrahepatic neutrophils cause liver injury in patients with AH, we used the mouse model of chronic-plus-binge alcohol feeding, known to be associated with high intrahepatic neutrophils (25, 34). In this study, we confirmed by IHC analyses an increase in intrahepatic neutrophils with minimal CD8^+^ T cell infiltration in the liver of ethanol-fed mice (Supplemental Figure 7); these findings were also supported by microarray analyses showing a significant increase in the expression of neutrophil-related but not CD8^+^ T cell–related genes in mice fed using the chronic-plus-binge ethanol model (Figure 6, C and D). Based on these results, we utilized this model for subsequent experiments to study the role of neutrophilic NCF1 in AH.

**Figure 7:**
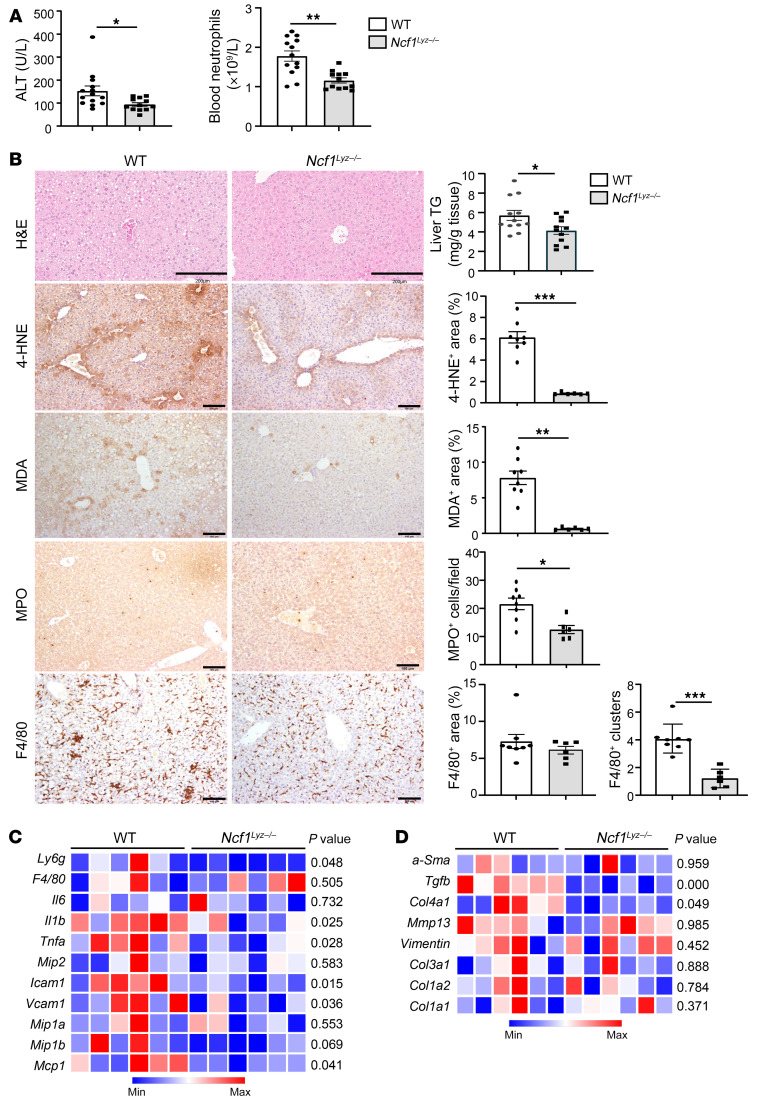
Genetic deletion of the *Ncf1* gene in neutrophils ameliorates chronic-plus-binge ethanol-induced liver ROS, inflammation, steatosis, and injury. WT and *Ncf1^Lyz–/–^* mice were fed an ethanol diet for 10 days, followed by gavage of ethanol, and were euthanized 9 hours later. (**A**) Serum ALT levels and circulating neutrophils were measured (WT, *n* = 14; KO, *n* = 12). (**B**) Liver tissues were subjected to H&E staining, TG measurement (WT, *n* = 12; KO, *n* = 12), and IHC staining (WT, *n* = 8; KO, *n* = 6) with antibodies against MDA, HNE, MPO, and F4/80. Representative images are shown on the left. Liver TG levels were quantified and are shown on the right. Quantification of the area positive for MDA or HNE staining is shown on the right. Scale bars: 200 μm (H&E row), 100 μm (additional rows). Quantification of number of MPO^+^ cells, percentage of F4/80^+^ area, and F4/80 clusters are on the right. Values in **A** and **B** represent mean ± SEM. *P < 0.05; **P < 0.01; ***P < 0.001. (**C** and **D**) Heatmap illustrations of the expression profiles of genes involved in inflammation (**C**) and fibrosis (**D**) (WT, *n* = 6; KO, *n* = 6). *P* values are indicated. Statistical significance was assessed using 2-tailed Student’s *t* test for comparing 2 groups (**A** and **B**) and 1-way ANOVA followed by Tukey’s post hoc test for multiple groups (**C** and **D**).

We previously reported that global *Ncf1*-deficient (*Ncf1*-KO) mice had lower serum ALT levels after chronic-plus-binge ethanol feeding (21). However, these mice developed significant weight loss and consumed less ethanol. To overcome these shortcomings and to specifically characterize the role of *Ncf1* in neutrophils, we generated myeloid cell–specific *Ncf1*-KO (*Ncf1^Lyz–/–^*) mice. The specific deletion of *Ncf1* in myeloid cells did not lead to the compensatory expression of other phagocytic oxidases, such as *Ncf2* and *Ncf4* (Supplemental Figure 8A). The *Ncf1^Lyz–/–^* mice were then subjected to chronic-plus-binge ethanol feeding. We found significantly lower levels of serum ALT, reduced total numbers of circulating neutrophils, and lower intrahepatic triglyceride (TG) levels in ethanol-fed *Ncf1^Lyz–/–^* mice compared with ethanol-fed WT mice (Figure 7A). H&E staining and liver TG measurement revealed that hepatic steatosis was ameliorated in ethanol-fed *Ncf1^Lyz–/–^* mice compared with WT mice (Figure 7B). The absence of neutrophilic *Ncf1* led to a marked decrease in the level of hepatic ROS, as determined by IHC staining for MDA and 4-HNE, by more than 90% in ethanol-fed *Ncf1^Lyz–/–^* mice compared with WT mice fed with ethanol (Figure 7B). Moreover, compared with ethanol-fed WT mice, ethanol-fed *Ncf1^Lyz–/–^* mice showed a lower number of intrahepatic MPO^+^ neutrophils, but had a comparable number of F4/80^+^ macrophages (Figure 7B). The distribution of some F4/80^+^ macrophages appeared to be in a cluster, representing infiltrating and activated macrophages, in ethanol-fed WT mice. However, in ethanol-fed *Ncf1^Lyz–/–^* mice, F4/80^+^ macrophages were more evenly distributed (Figure 7B), suggesting that ethanol-fed *Ncf1^Lyz–/–^* mice have lower levels of activated macrophages in the liver than ethanol-fed WT mice. *Ncf1^Lyz–/–^* mice fed with ethanol also demonstrated a significant decrease in the expression of several inflammatory mediators (*Il1b*, *Tnfa*, *Icam1*, *Mip1b*, and *Ly6g*) and fibrosis-associated genes (*Tgfb* and *Col4a1*) when compared with ethanol-fed WT mice (Figure 7, C and D, and Supplemental Figure 8, B and C).

### Neutrophil-specific NCF1 promotes alcohol-induced steatosis by inhibiting hepatic SIRT1 and AMPK activation.

**Figure 8:**
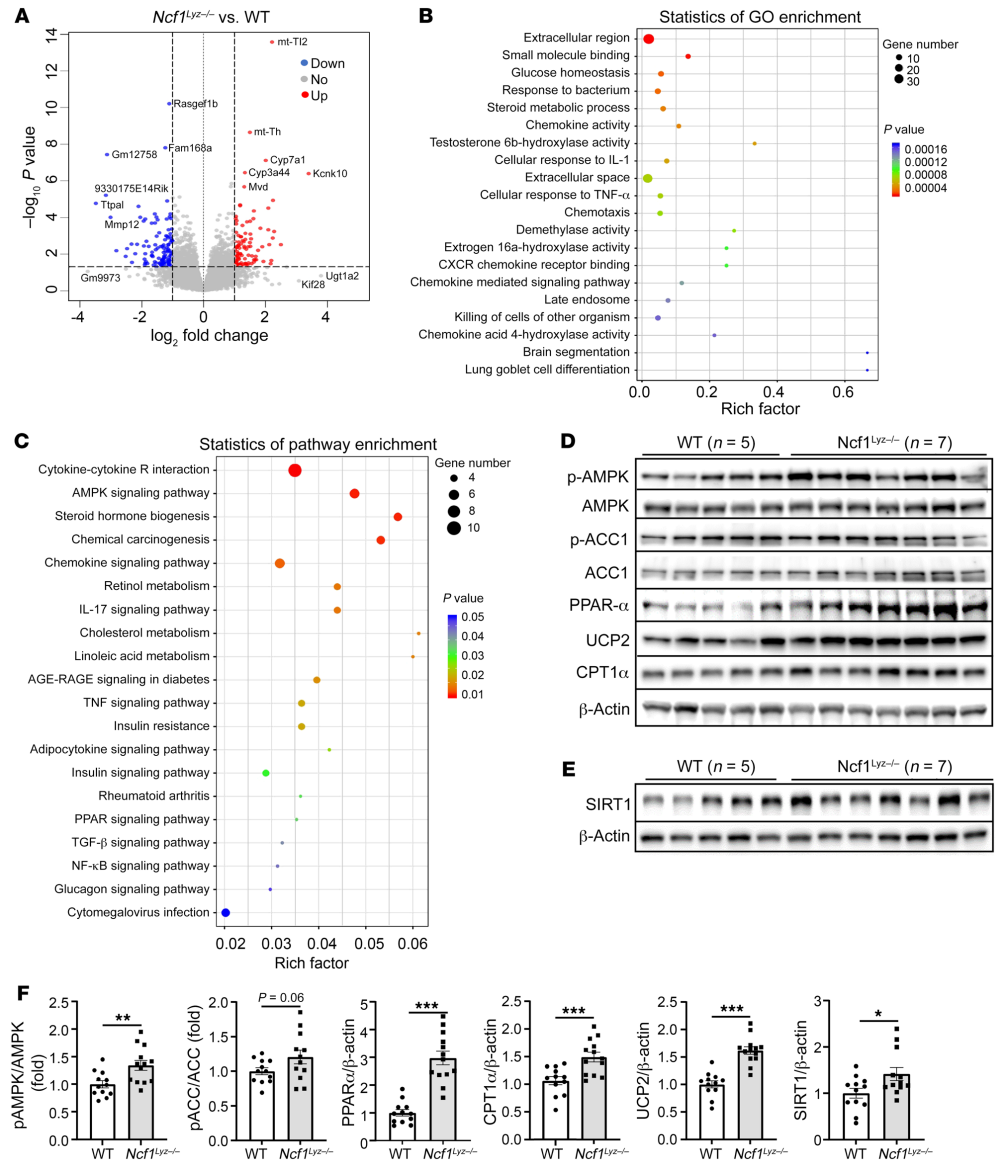
Neutrophil-specific NCF1 promotes alcoholic steatosis by inhibiting hepatic SIRT1 and AMPK activation. RNA-Seq was performed in ethanol-fed WT (*n* = 5) and *Ncf1^Lyz–/–^* (*n* = 4) mouse livers. (**A**) Volcano plot displaying DEGs between WT and *Ncf1^Lyz–/–^* mice. (**B** and **C**) Analyzed DEGs were subjected to GO and KEGG pathway enrichment analysis. The top 20 enriched GO terms and KEGG pathways are shown in **B** and **C**, respectively. (**D**–**F**) Liver tissues from ethanol-fed mice (WT, *n* = 5; KO, *n* = 7) were subjected to immunoblot analysis of p-AMPK, p-ACC, PPARα, UCP2, CPT1α, and SIRT1 (**D** and **E**). Quantification of protein band densities from 2 independent experiments (**D** and **E** and Supplemental Figure 9, C and D) is shown in **F**. Data are represented as mean ± SEM. *P < 0.05; **P < 0.01; ***P < 0.001. Statistical significance was assessed using 2-tailed Student’s *t* test for comparing 2 groups (**F**).

To identify the mechanisms mediating the effect of neutrophil-specific NCF1 on alcohol-induced hepatic steatosis, inflammation, and fibrosis, we performed RNA-Seq analyses of the liver tissues from ethanol-fed WT and *Ncf1^Lyz–/–^* mice. Using the differential expression cutoff of log_2_ (fold change) higher than 1 or lower than –1 and *P* < 0.05, we identified 98 and 126 genes that were up- and downregulated in ETOH-fed *Ncf1^Lyz–/–^* compared with WT mice, respectively (Supplemental Figure 9A). A volcano plot of DEGs is illustrated in Figure 8A. We next generated a heatmap of the top 100 DEGs based on the *P* value (Supplemental Figure 9B). The detailed analyses of the top 20 enriched GO terms and Kyoto Encyclopedia of Genes and Genomes (KEGG) (https://www.genome.jp/kegg/) pathways are shown in Figure 8, B and C; one of the enriched pathways is related to AMPK signaling (Figure 8C).

AMPK is a metabolic sensor that controls lipid metabolism (26). Upon its activation, AMPK phosphorylates acetyl co-A carboxylase (ACC), leading to the inhibition of ACC activity and a decrease in fatty acid synthesis. NAD^+^-dependent deacetylase SIRT1 regulates hepatocyte lipid synthesis through AMPK activation (35, 36). Western blot analyses revealed that ethanol-fed *Ncf1^Lyz–/–^* mice had a significant increase in hepatic phosphorylation of AMPK (p-AMPK) and its downstream substrate, phospho-ACC (p-ACC), PPARα, carnitine palmitoyltransferase-1α (CPT1α),and uncoupling protein 2 (UCP2) as well as SIRT1 when compared with ethanol-fed WT mice (Figure 8, D–F, and Supplemental Figure 9, C and D). Taken together, our data suggest that neutrophil-specific NCF1 deficiency reduces alcoholic steatosis by enhancing hepatic AMPK and SIRT1 activation.

### Neutrophilic NCF1 promotes liver inflammation and fibrosis by inhibiting antiinflammatory and antifibrotic miR-223 in ROS and p38 MAPK-dependent manners.

**Figure 9:**
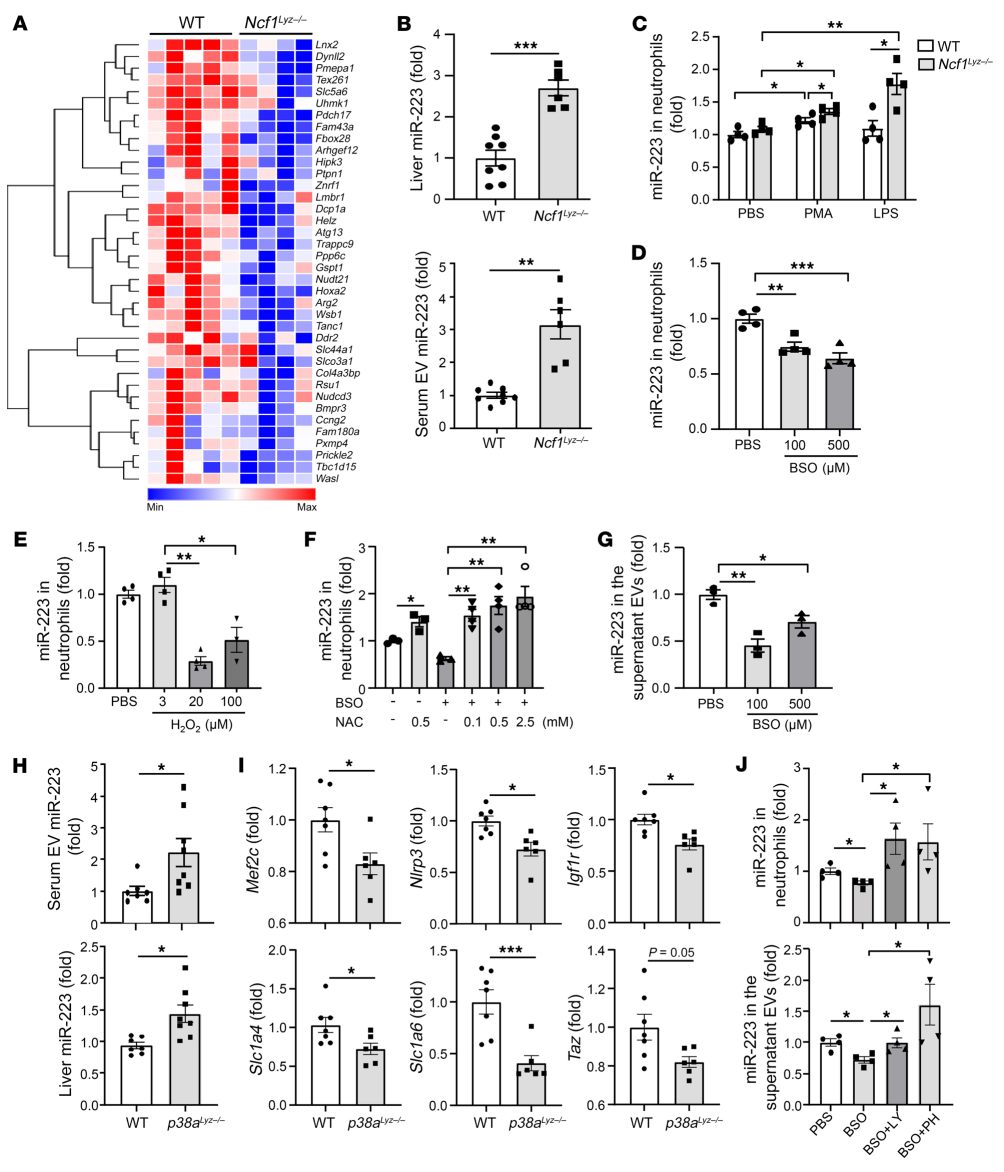
Neutrophilic NCF1 promotes liver inflammation and fibrosis by inhibiting antiinflammatory and antifibrotic miR-223 in ROS and p38 MAPK–dependent manners. (**A**) Heatmap analysis of miR-223 target genes in ethanol-fed WT (*n* = 5) and *Ncf1^Lyz–/–^* (*n* = 4) mice based on RNA-Seq data. (**B**) miR-223 levels in serum EVs and liver were measured in ethanol-fed WT (*n* = 8) and *Ncf1^Lyz–/–^* (*n* = 6) mice. (**C**) Neutrophils were isolated from WT and *Ncf1^Lyz–/–^* mice and treated with LPS (50 ng/ml) or PMA (30 ng/ml) for 6 hours. miR-223 in the neutrophils was measured (*n* = 4 per group). (**D** and **E**) Neutrophils were isolated from WT mice and treated with BSO (**D**) or H_2_O_2_ (**E**). miR-223 expression in the neutrophils was measured (*n* = 4 per group). (**F**) Neutrophils were isolated from WT mice and treated with BSO at 0 or 100 μM with or without NAC treatment. miR-223 expression in the neutrophils was measured (*n* = 4 per group). (**G**) Neutrophils were isolated from WT mice and treated with BSO. miR-223 expression in the supernatant EVs was measured (*n* = 3 per group). (**H** and **I**) WT and *p38a^Lyz–/–^* mice were fed an ethanol diet for 10 days, followed by gavage of ethanol, and were euthanized 9 hours later. miR-223 levels in serum EVs and liver were measured (**H**) (WT, *n* = 7; KO, *n* = 8). miR-223 target genes in the liver were examined by real-time qPCR (**I**) (WT *n* = 7; KO *n* = 6). (**J**) Neutrophils were isolated from WT mice and treated with BSO at 100 μM with or without p38α inhibitors LY2228820 (2 μM) or PH797804 (4 μM). miR-223 expression in the neutrophils and supernatant EVs was measured (*n* = 4 per group). Data are represented as mean ± SEM. *P < 0.05; **P < 0.01; ***P < 0.001. Statistical significance was assessed using 2-tailed Student’s *t* test for comparing 2 groups (**B**, **C**, **H**, and **I**) and 1-way ANOVA followed by Tukey’s post hoc test for multiple groups (**D**, **G**, and **J**).

RNA-Seq analysis showed that genes associated with inflammatory and fibrotic pathways were enriched in the top 100 DEG, as shown in Figure 8, B and C. A large number of these genes were downregulated in ethanol-fed *Ncf1^Lyz–/–^* mice compared with WT mice fed with ethanol as shown in Supplemental Figure 9B. We previously reported that neutrophilic miR-223 exerts antiinflammatory and antifibrotic effects (28–30). Interestingly, we observed that many miR-223–targeted genes, which are negatively regulated by miR-223, were downregulated in ethanol-fed *Ncf1^Lyz–/–^* mice compared with WT mice (Figure 9A). In agreement with the reduction in the expression of several miR-223–targeted genes in *Ncf1^Lyz–/–^* mice, we found an increase in hepatic miR-223 levels in these mice (Figure 9B). In addition, we also found an increase in circulating extracellular vesicle–associated (EV-associated) miR-223 in ethanol-fed *Ncf1^Lyz–/–^* mice (Figure 9B).

An increase in ROS generation by NCF1 led us to hypothesize that NCF1-derived ROS may regulate miR-223 expression in neutrophils. To test this hypothesis, we performed in vitro experiments using the following approaches. First, we treated neutrophils isolated from WT and *Ncf1^Lyz–/–^* mice with LPS or PMA; both are known for activating neutrophils and ROS production via NCF1. We observed a significant decrease of ROS production as well as a remarkable increase in the expression of miR-223 in neutrophils from *Ncf1^Lyz–/–^* mice treated with either PMA or LPS when compared with those from WT mice (Supplemental Figure 10A and Figure 9C). Second, we treated neutrophils with 2 ROS inducers, hydrogen peroxide (H_2_O_2_) and DL-buthionine-(S, R)-sulfoximine (BSO), an inhibitor of glutathione biosynthesis. We found a significant reduction in neutrophilic miR-223 expression when they were treated with either BSO or H_2_O_2_ (Figure 9, D and E). We next treated neutrophils with BSO in the presence and absence of ROS scavenger N-acetylcysteine (NAC) and observed that the inhibitory effect of BSO on miR-223 expression was diminished in the presence of NAC (Figure 9F), confirming our hypothesis on the regulation of miR-223 by ROS. The ROS induction not only inhibited the expression of neutrophilic miR-223; it also reduced the release of EV-associated miR-223 by neutrophils (Figure 9G).

We next asked how ROS regulates neutrophilic miR-223 expression. Once generated, ROS activates stress kinases such as p38 MAPK, which was confirmed by the following experiments. First, ethanol-fed *Ncf1^Lyz–/–^* mice had lower hepatic levels of p-p38 MAPK compared with ethanol-fed WT mice (Supplemental Figure 10B). Second, treatment with H_2_O_2_ upregulated the phosphorylation of p38 MAPK in neutrophils (Supplemental Figure 10C). Furthermore, in vitro experiments showed that p38 MAPK activation induced by PMA was markedly suppressed in neutrophils from *Ncf1^Lyz–/–^* mice compared with those from WT mice (Supplemental Figure 10D).

To test the hypothesis that ROS-induced neutrophilic miR-223 expression is mediated by p38 MAPK, we generated myeloid cell–specific *p38a*-KO (*p38a^Lyz–/–^*) mice and performed chronic-plus-binge ethanol feeding. As shown in Figure 9H, we observed not only an increase in hepatic miR-223, but also serum-derived EV-associated miR-223 expression in ethanol-fed *p38a^Lyz–/–^* mice compared with ethanol-fed WT mice. In parallel with an increase in miR-223 expression, the expression of known miR-223 target genes *Mef2c*, *Nlrp3*, *Igf1r*, *Slc1a6*, and *Slc1a4* was markedly reduced in ethanol-fed *p38a^Lyz–/–^* mice (Figure 9I). In vitro experiments showed that treatment with the p38 inhibitor LY or PH can rescue miR-223 expression downregulated by BSO in neutrophils as well as in neutrophil-derived EVs (Figure 9J). Additionally, we found that p38 inhibitor significantly increased EV release from neutrophils, suggesting the inhibitory effect of p38 on miR-223 expression, which acts by downregulating EV production in neutrophils (Supplemental Figure 10E). Finally, the lack of neutrophilic p38 MAPK significantly ameliorated ethanol-induced liver injury, as indicated by a reduction of serum ALT levels, hepatic ROS levels, and expression of proinflammatory genes (Supplemental Figure 11, A–D).

### Hepatic miR-223 is highly elevated in SAH patients and positively correlates with the number of neutrophils in the liver.

**Figure 10:**
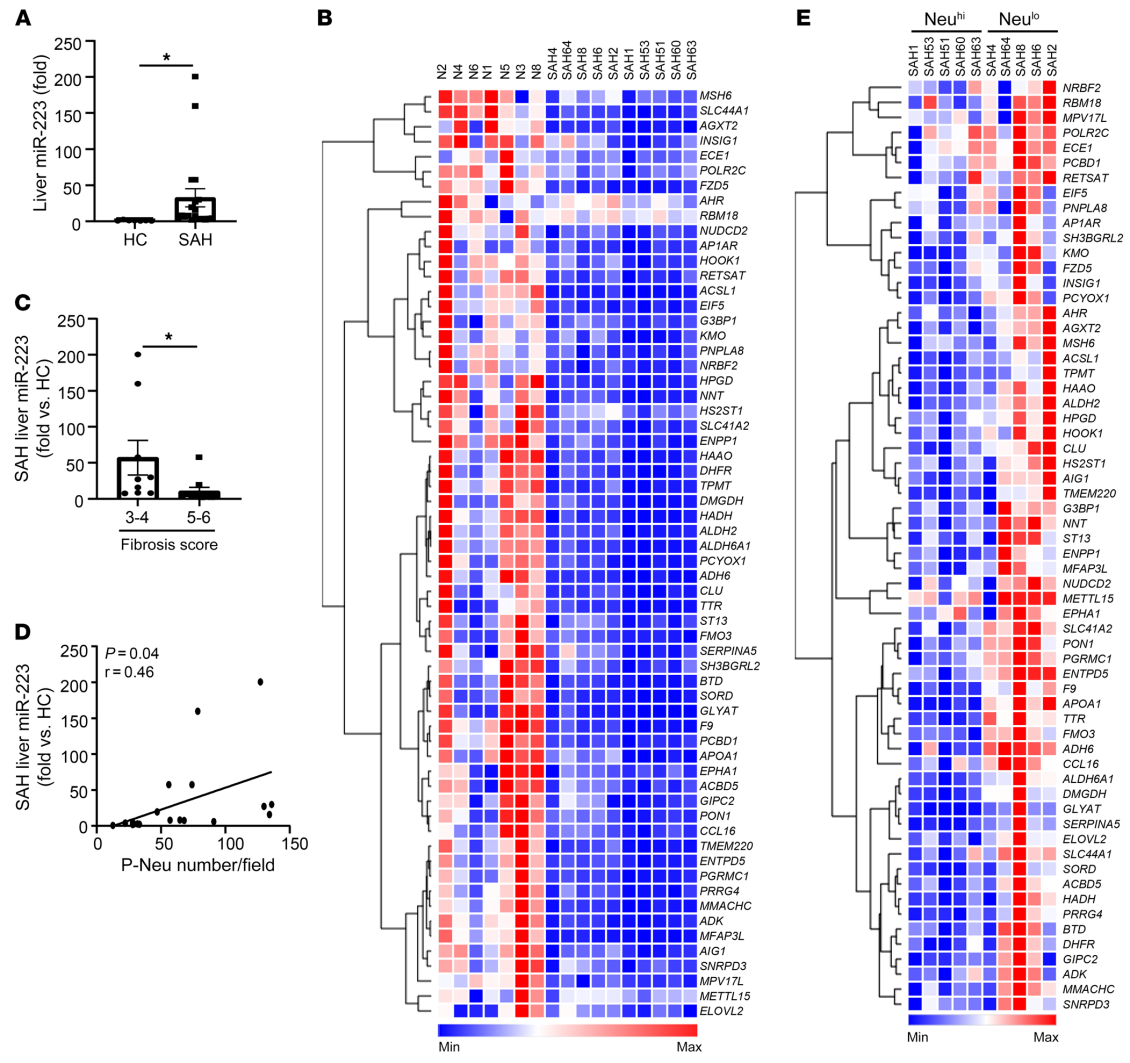
Hepatic miR-223 is highly elevated in SAH patients and positively correlated with the number of neutrophils in the liver. (**A**) Hepatic miR-223 levels were compared between healthy control (*n* = 7) and SAH patients (*n* = 20) by RT-qPCR. (**B**) Heatmap analysis of miR-223 target genes in the liver among healthy controls (*n* = 7) and SAH patients (*n* = 10) based on RNA-Seq data. (**C**) Hepatic miR-223 levels were compared among SAH patients with low (*n* = 10) or high (*n* = 10) fibrosis score (refer to Figure 4B). (**D**) Correlation analysis was performed between hepatic miR-223 level and P-Neu number/field (*n* = 20). (**E**) Heatmap analysis of miR-223 target genes in the liver of SAH patients (*n* = 10). Data are represented as mean ± SEM. *P < 0.05. Statistical significance was assessed using 2-tailed Student’s *t* test for comparing 2 groups (**A** and **C**) and Pearson’s correlation analysis (**D**).

To translate the observations in our mouse models to humans, we next determined the expression of hepatic miR-223 in SAH patients. We found a significant increase in hepatic miR-223 expression (Figure 10A) that coincided with the downregulation of its target genes in patients with SAH compared with controls (Figure 10B). The expression of hepatic miR-223 was lower in SAH patients with advanced fibrosis, stage 5 or 6, compared with those in stage 3 or 4 (Figure 10C); its expression also positively correlated with the number of neutrophils in the parenchyma (Figure 10D). Finally, the expression of miR-223 target genes was significantly lower in the livers of Neu^hi^ SAH patients when compared with those of Neu^lo^ (Figure 10E). Additionally, we conducted correlation analysis and observed a positive correlation between hepatic *NCF1* and miR-223 (Supplemental Figure 12).

## Discussion

**Figure 11:**
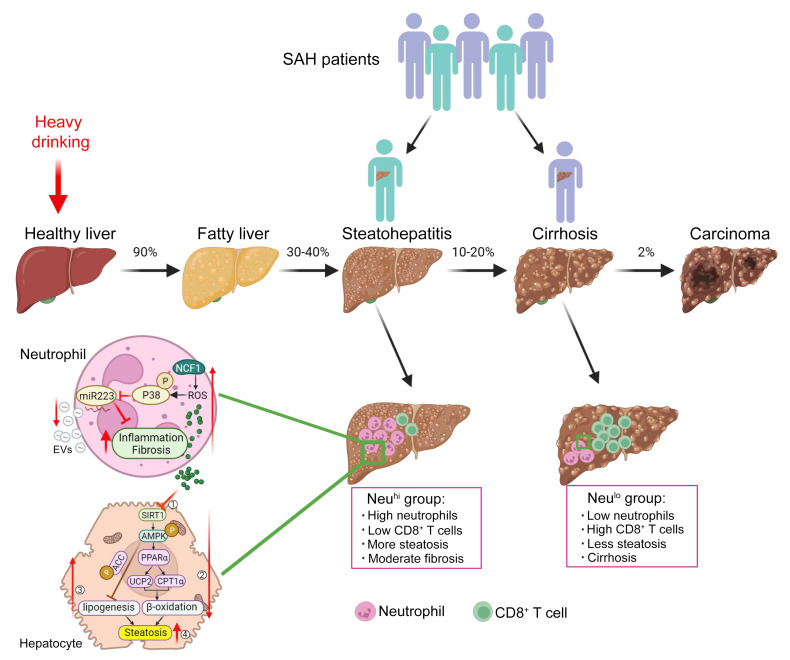
Model depicting distinct histopathological phenotypes in SAH patients based on liver immune phenotyping and the role of neutrophilic NCF1-dependent ROS in AH pathogenesis. SAH exists as 2 distinct histopathological phenotypes, Neu^hi^CD8^lo^ and Neu^lo^CD8^hi^, suggesting different pathogenesis leading to liver injury and/or failure among these patients. Patients with high hepatic neutrophils but low CD8^+^ T cells are younger and have higher MELD scores and ALT levels, but have less fibrosis compared with those with Neu^lo^CD8^hi^. Among those with Neu^hi^, neutrophilic NCF1-dependent ROS promotes AH progression through the NCF1/SIRT1/AMPK axis on lipid metabolism and NCF1/p38 MAPK/miR-223 on alcohol-induced inflammation and fibrosis.

Patients with AH have a wide range of clinical presentation, disease severity, and overall prognosis. In mild cases, patients may improve with conservative management and have a favorable outcome (37). However, in those with severe presentation, such as SAH, the disease is characterized by high short-term mortality (38) and early liver transplantation is required (39). In our current study, we observed two distinct hepatic phenotypes among explanted SAH livers, based on histology and the extent of inflammatory cell infiltration, Neu^hi^CD8^lo^ and Neu^lo^CD8^hi^, suggesting different pathogenesis leading to liver injury and/or failure among these patients (Figure 11). The intervariability of neutrophil infiltration has also been reported in a study using ^111^In-leukocyte scintigraphy to image hepatic neutrophil migration in patients with AH (40). Patients with high hepatic neutrophils but low CD8^+^ T cells were younger and had higher MELD scores and ALT levels, but had less fibrosis compared with those with Neu^lo^CD8^hi^. Neutrophils likely play a more important role in promoting disease progression and liver failure in SAH with Neu^hi^CD8^lo^ than those with Neu^lo^CD8^hi^. The design of our study precludes the exploration of what triggers the differential phenotypes or whether these phenotypes represent the two extremes of a continuum among patients with SAH; these questions deserve further study. Our results have substantial clinical implications, as the findings suggest that different treatments are required for these two SAH populations.

Our data revealed that the number of intrahepatic neutrophils correlated with MELD scores and serum ALT levels in SAH patients and that neutrophil-derived ROS promoted liver injury and inflammation in the experimental model. This suggests that neutrophil-generated ROS is one of the mechanisms that contributes to liver injury and dysfunction in SAH in addition to many other cell types involved in SAH pathogenesis (12). Regardless of the underlying process, MELD is an objective clinical parameter and can be used as a surrogate for the severity of underlying liver disease. Our finding that the abundance of neutrophils correlated with MELD scores does not imply that the changes in MELD are mainly caused by neutrophil-generated ROS. Rather, the observation provided a scientific rationale for the inflammatory process driven by the differential level of hepatic neutrophil infiltration correlating with underlying disease severity. Interestingly, previous studies reported that a high level of fibrosis is associated with a worse prognosis in AH patients, whereas greater neutrophil number is associated with better survival in AH patients (32, 41, 42). However, in the present study, all SAH patients suffered from end-stage liver failure and were subjected to early transplantation, so these SAH cohorts are not suitable for analyzing the correlation between the degree of fibrosis and prognosis.

The presence of hepatic neutrophils is strongly associated with activation of inflammatory pathways and ROS production, as illustrated by our RNA-Seq analysis stratified by the degree of hepatic neutrophil infiltration. Among several neutrophilic genes, the expression of neutrophilic *NCF1* was significantly higher in SAH patients with high hepatic neutrophil infiltration. Its expression is closely associated with inflammation and, as expected, oxidative stress–related genes not only in patients with SAH, but also in those with alcoholic steatohepatitis. To further explore the mechanistic insight on the role of neutrophilic NCF1 in liver injury in patients with AH, we generated myeloid-specific *Ncf1*-KO (*Ncf1^Lyz–/–^*) mice and tested them in a mouse model of chronic-plus-binge ethanol feeding, a model known to be associated with significant intrahepatic neutrophil infiltration (25). Our data revealed that the deletion of the *Ncf1* gene in myeloid cells markedly ameliorated hepatic ROS production, steatosis, and inflammation in the chronic-plus-binge ethanol-feeding model. The important role of myeloid NCF1 in inducing liver ROS was also previously reported in a mouse model of NAFLD (43). Furthermore, our immunohistochemistry analyses revealed that NCF1 expression was restricted to MPO^+^ neutrophils in mouse livers. As NCF1 is known to be expressed predominantly in neutrophils (24), the NCF1 effects we observed in myeloid-specific *Ncf1*-KO mice are mainly from neutrophils.

### Role of neutrophilic NCF1/SIRT1/AMPK in alcohol-induced steatosis in AH.

Given the potential role of neutrophil-generated ROS in SAH pathogenesis, we studied the underlying mechanisms in a mouse AH model of chronic-plus-binge ethanol feeding. RNA-Seq analysis revealed the AMPK pathway is one of the top pathways inhibited by neutrophilic NCF1-generated ROS, which may contribute to steatosis development. AMPK, a serine-threonine kinase, regulates cellular homeostasis (35). Once activated by phosphorylation, it stimulates energy-producing processes, such as fatty acid oxidation, and inhibits energy-utilizing processes, such as fatty acid synthesis (44). Inhibition of AMPK by alcohol feeding has been shown to contribute to alcohol-induced hepatic steatosis (26). We also found that the AMPK upstream activator SIRT1 as well as several AMPK downstream target proteins, such as PPARα, UCP2, and CPT1α, all of which control hepatic fatty acid oxidation, were upregulated in ethanol-fed *Ncf1^Lyz–/–^* mice compared with WT mice. In addition, it has been well established that the expression and activity of SIRT1 can be suppressed by ROS in many cell types (45, 46), which is in agreement with our finding that SIRT1 expression was highly elevated in *Ncf1^Lyz–/–^* mice in the ALD model. Moreover, a substantial body of evidence suggests that upregulated SIRT1 can cause activation of AMPK through LKB1 in several types of tissues (47). Our results suggest that the reduction of ROS production secondary to the absence of neutrophilic NCF1 leads to hepatic activation of SIRT1 and AMPK, shifts the intracellular lipid metabolism toward fatty acid oxidation, and ameliorates alcohol-induced hepatic steatosis.

### Role of the neutrophilic NCF1/p38 MAPK/miR-223 axis in alcohol-induced inflammation and fibrosis in AH.

We previously reported that genetic deletion of the miR-223 gene exacerbated ethanol-induced hepatic injury, neutrophil infiltration, ROS, and upregulated hepatic expression of p47^phox^ (21), suggesting that miR-223 plays an important role in inhibiting liver inflammation. miR-223 has also been shown to block hepatic fibrosis (30, 48, 49). Interestingly, our RNA-Seq data indicate significant downregulation of several miR-223–targeted genes associated with inflammation and fibrosis (28–30) in ethanol-fed *Ncf1^Lyz–/–^* mice. This prompted us to hypothesize that lack of neutrophilic NCF1 and ROS can activate neutrophilic miR-223 expression. Neutrophilic miR-223 expression in *Ncf1^Lyz–/–^* mice that had lower ROS levels was significantly higher compared with that of WT mice. In vitro treatment of neutrophils with LPS, PMA (to generate ROS production), or ROS inducers caused a significant reduction in neutrophilic miR-223, suggesting that ROS inhibits miR-223 expression in neutrophils. On the upstream, our results suggest that the inhibitory effect of NCF1-induced ROS on miR-223 is mediated by p38 MAPK, as demonstrated by using p38 MAPK inhibitors or *p38a^Lyz–/–^* mice. Taken together, our data provide a mechanistic link on the role of the neutrophilic NCF1/p38 MAPK/miR-223 axis in regulating hepatic inflammation and fibrosis through miR-223 target genes.

### Clinical and mechanistic implications of this study.

In conclusion, we reported two distinct histopathological phenotypes of SAH patients based on the quantitative analysis of inflammatory cell infiltration, which may help distinguish subpopulations of SAH patients who cannot be defined by standard lab tests. The varying degree of hepatic neutrophil infiltration in SAH patients may have differential effects in driving downstream liver injury and liver failure through the inflammatory process and ROS production. This observation likely underlies the heterogeneity in response to corticosteroids in various clinical studies. Our results also call for a search for biomarkers as surrogate indicators for these two distinct hepatic phenotypes and provide a rationale for future therapeutic trials, which may need to be personalized based on the pattern of inflammatory cell infiltration. Mechanistically, we expanded existing knowledge on the roles of hepatic neutrophils in mediating liver injury by highlighting the effects of the NCF1/SIRT1/AMPK axis on lipid metabolism and the NCF1/p38 MAPK/miR-223 pathway on alcohol-induced inflammation and fibrosis (Figure 11). Although our data uncovered an important mechanism underlying steatosis development, the presence of steatosis itself may not be directly associated with AH severity and MELD score. Since the mouse model is associated with mild liver injury and inflammation, we were unable to uncover the other pathways that drive high MELD scores that are used to grade liver dysfunction severity in AH patients.

## Methods

### Human liver samples: healthy controls.

We obtained unstained paraffin-embedded healthy control liver tissues (total *n* = 19) from the Liver Tissue Cell Distribution System (Minneapolis, Minnesota) (supported by the NIH, HHSN276201200017C), the Department of Surgery at the Johns Hopkins Hospital (supported by the R24AA025017, Clinical resources for alcoholic hepatitis investigators), and the Department of Pathology and Laboratory Medicine, Indiana University School of Medicine.

### SAH samples from explanted livers.

Frozen liver tissues and unstained paraffin-embedded liver tissues (total *n* = 40) from the explanted livers of patients with SAH were provided by the Department of Surgery at the Johns Hopkins Hospital. Baseline demographics, clinical characteristics, and laboratory tests of SAH patients are shown in Supplemental Table 1. Baseline demographics, clinical characteristics, and laboratory tests of SAH patients stratified by sex are shown in Supplemental Table 2.

*Liver biopsy samples from alcoholic steatohepatitis*: Unstained paraffin-embedded liver biopsy tissues from patients with alcoholic steatohepatitis (total *n* = 12) were obtained from the Department of Pathology and Laboratory Medicine, Indiana University. Baseline demographics, clinical characteristics, and laboratory tests of these patients are shown in Supplemental Table 3.

### Alcoholic cirrhosis samples.

Unstained paraffin-embedded liver tissues from explanted livers of patients with alcoholic cirrhosis (total *n* = 33) were obtained from the Liver Tissue Cell Distribution System.

### Mouse models.

*Ncf1*-floxed mice were generated using CRISPR/Cas9 technology by inserting 2 LoxP sequences into intron 1 and intron 8 of the mouse *Ncf1* gene (Applied StemCell). The *p38a*-floxed mice were provided by Yibin Wang (David Geffen School of Medicine, University of California, Los Angeles, California, USA). The background of *Ncf1*-floxed mice was on C57BL/6J. *Ncf1*-floxed and *p38a*-floxed mice were then crossed with Lyz-Cre mice (Jackson Laboratory) to generate myeloid cell–specific *Ncf1*-KO (*Ncf1^Lyz–/–^*) mice and *p38a*-KO (*p38a^Lyz–/–^*) mice via several steps, respectively. We used Lyz-Cre–negative *Ncf1*-floxed mice and *p38a*-floxed mice as their corresponding WT littermate controls. Mice were housed in a temperature-controlled room with a 12-hour dark/12-hour light cycle.

The chronic-plus-binge ethanol-feeding model was described previously (34, 50). Briefly, 10- to 12-week-old female mice were initially fed with Lieber-DeCarli liquid diet (catalog F1259SP, Bio-Serv) ad libitum for 5 days during acclimatization. Subsequently, the ethanol-fed groups were allowed free access to an ethanol diet (catalog F1258SP, Bio-Serv) containing 5% (v/v) ethanol for 10 days. On day 11, they received a single dose of ethanol (5 g/kg body weight) via oral gavage in the early morning and were sacrificed 9 hours later. Pair-fed mice were fed with an isocaloric control diet for 10 days, followed by an oral gavage with isocaloric dextrin-maltose on the day of sacrifice.

### Biochemical assays.

Serum ALT levels were determined from serum collected from mouse retroorbital plexus, using Catalyst Dx Chemistry Analyzer (IDEXX Laboratories) according to the manufacturer’s instructions.

### Hepatic TG measurement.

Liver tissues (~50 mg) were homogenized in 300 μL chloroform:methanol (1:2 volume) for 2 minutes followed by a second homogenization for 30 seconds with the addition of 300 μL of chloroform. The homogenates were mixed with 100 μl of water and rehomogenized for 30 seconds. The lipid layer (~600 μL) was separated via centrifugation at 800*g* for 10 minutes at room temperature. The lower phase, enriched in lipid, was transferred and dried. The lipid extract was suspended in 300 μL of 5% Triton X-100 PBS, pH 7.4. The measurement was performed using Pointe Scientific Triglycerides Liquid Reagents according to the manufacturer’s instructions.

### Liver histology, immunohistochemistry, and sequential multiplex immunofluorescence staining.

Liver specimens were fixed in 10% buffered formalin and embedded in paraffin. Then 4 μm sections were cut and stained with Sirius red dyes (MilliporeSigma) or for immunohistochemistry staining with specific antibody. For immunohistochemistry, after heat-induced epitope retrieval, paraffin-embedded sections were incubated in 3% H_2_O_2_ and blocked in 3% normal serum buffer. Sections were incubated with primary antibodies overnight at 4°C. Vectastain Elite ABC Staining Kit and DAB Peroxidase Substrate Kit (Vector Laboratories) were used to visualize the staining according to the manufacturer’s instructions. The following primary antibodies were used: MPO (catalog PP 023 AA, H) from Biocare Medical; F4/80 (catalog 70076S) from Cell Signaling Technology; CD68 (catalog M087601-2), CD8 (catalog GA62361-2), and CD20cy (catalog M075501-2) from Dako; CD3 (catalog ab16669), CD4 (catalog ab183685), FOXP3 (catalog ab20034), and IL-17 (catalog ab79056) from Abcam; NCF1 (catalog PA5-36863) from Thermo Fisher; and MDA (clone 1F83) and 4-HNE (clone HNE-J2) from Genox. Positive cells and positive areas in 5 to 10 randomly selected high-power fields were analyzed. For immunofluorescence staining, after primary antibody incubation overnight, cyanine-conjugated secondary antibodies, such as Cy2 donkey anti-goat IgG (H+L) (Jackson ImmunoResearch) or Cy3 anti-mouse or rabbit IgG (H+L) (Jackson ImmunoResearch) were incubated for 30 minutes at room temperature. Slides were incubated with DAPI (Vector Laboratories) for 5 minutes for nuclear staining. Images were obtained using Leica AF6000 Modular Systems.

Sequential multiplex immunofluorescence staining on formalin-fixed, paraffin-embedded liver sections was performed, as previously described (51, 52). Images were acquired on a Zeiss AxioObserver 7. Acquired images were processed and analyzed using FIJI (53), Ilastik (version 1.3.3post3) (54), and CellProfiler (version 4.1.3) (55). Cellular distribution was represented using FlowJo Software for Windows, version 10.6.1 (BD, 2019). The following antibodies were used: HepPar1 (catalog M7158, Dako), CD8a (catalog NBP2-34318-0.1 mg, Novus), CD4 (catalog ab133616, Abcam), MPO (catalog ab208670, Abcam), CD20 (catalog PA5-16701, Thermo Fisher), IBA1 (catalog MABN92, EMD Millipore), and α-SMA (catalog M085129-2, Agilent).

### Isolation and culture of mouse neutrophils.

Bone marrow neutrophils were collected from *Ncf1^fl/fl^* and *Ncf1^Lyz–/–^* mice using a mouse Neutrophil Isolation Kit (Miltenyi Biotec) based on the manufacturer’s instructions and then cultured in RPMI-1640 containing 10% EV-free FBS and penicillin-streptomycin. In some experiments, neutrophils were cultured with LPS, PMA, or BSO plus 2 μM LY2228820 (p38 MAP kinase inhibitor) or 4 μM PH797804 (p38 MAPK inhibitor) for 6 hours. All inhibitors were obtained from Selleck Chemicals. In certain experiments, neutrophils were cultured with H_2_O_2_ for 5 minutes, 15 minutes, 30 minutes, 1 hour, or 2 hours.

### Flow cytometry analyses of ROS levels in neutrophils.

Bone marrow neutrophils were collected from *Ncf1*^fl/fl^ and *Ncf1^Lyz–/–^* mice using the Mouse Neutrophil Isolation Kit (Miltenyi Biotec) based on the manufacturer’s instructions. Neutrophils were resuspended in prewarmed medium (RPMI-1640 containing 10% FBS and penicillin-streptomycin) containing the probe H2DCFDA (Thermo Fisher) at the working concentration of 20 μM and incubated for 30 minutes at 37°C. Then 100 ng/ml PMA or 100 ng/ml LPS was added into the medium and incubated for an additional 30 minutes at 37°C. H2DCFDA intensity was measured by flow cytometry (CytoFLEX, Beckman Coulter) in the FL1 channel.

### EV isolation.

EVs were isolated as previously described (56). Briefly, after centrifugation of serum or cultured medium at 3000*g* for 15 minutes at 4°C, the supernatant fraction including EVs was incubated with an appropriate volume of ExoQuick or ExoQuick-TC exosome precipitation solution (System Biosciences), depending on the type of samples for EV isolation procedure, according to the manufacturer’s instructions. The number of EVs in the supernatant was measured by NanoSight LM14C.

### Total RNA isolation and RT-qPCR.

Total RNA was purified from liver tissues or cell cultures using TRIzol Reagent (Thermo Fisher) according to the manufacturer’s instructions. RNA (1 μg of total) was reverse transcribed into cDNA using a high-capacity cDNA Reverse Transcription Kit (Thermo Fisher). The expression levels of mRNA were measured by reverse-transcription quantitative PCR (RT-qPCR) with an ABI7500 RT-PCR system (Applied Biosystems). *8s* rRNA was used as the invariant housekeeping control gene. The 2^−ΔΔCt^ method was used to calculate relative gene expression. The primers used for RT-qPCR are listed in Supplemental Table 4.

For miRNA-223 detection, RNA samples were purified and reverse transcribed using the TaqMan microRNA Reverse Transcription Kit (Applied Biosystems). Subsequently, mature miRNA and pri-miRNA expression were amplified by using TaqMan miRNA Assay Kits (Applied Biosystems) and TaqMan Pri-miRNA Assays (Applied Biosystems) according to the manufacturer’s protocols. SnoRNA202 or Spike-In cel-miR-39 was used as an internal control.

### Western blotting.

Liver tissues and cells were homogenized or lysed in RIPA buffer containing a cocktail of protease inhibitors (Santa Cruz Biotechnology Inc.) according to the manufacturer’s instructions, then vortexed and centrifuged at 10,000*g* at 4°C for 10 minutes. Protein extracts were mixed in Laemmli loading buffer, boiled for 10 minutes, and then subjected to SDS-PAGE. After electrophoresis on 4% to 12% Bis-Tris gel (Bio-Rad), proteins were transferred onto nitrocellulose membranes (Thermo Fisher) and blotted against respective primary antibodies overnight. Antibodies against p-p38 (Thr180/Tyr182, catalog 4511S), p38 (catalog 8690S), p-ACC (catalog 3661S), ACC (catalog 3676S), p-AMPK (catalog 50081S), AMPK (Cat# 2532L), SIRT1 (catalog 9475S), UCP2 (catalog 89326S), and β-actin (catalog 3700S) were purchased from Cell Signaling Technology; antibodies against CPT1 (catalog sc-393070) and PPARa (catalog sc-398394) were purchased from Santa Cruz Biotechnology Inc.; antibodies against p-ASK1 (Thr838, catalog PA5-118633) and ASK1 (catalog PA5-118392) were purchased from Thermo Fisher. Membranes were washed with 0.1% (v/v) Tween 20 in PBS (pH 7.4) and incubated with a 1:5000 dilution of horseradish peroxidase–conjugated secondary antibody for 1 hour. Protein bands were visualized by SuperSignal West Femto Maximum Sensitivity Substrate (Thermo Fisher).

### RNA-Seq and bioinformatics analysis.

The total RNAs were extracted from EtOH-fed 5 WT and 4 *Ncf1^Lyz–/–^* mouse livers and submitted to Poly(A) RNA Sequencing (LC Sciences). The library was prepared following Illumina’s TruSeq Stranded mRNA sample preparation protocol, and RNA integrity was checked with Agilent Technologies 2100 Bioanalyzer. Only RNA samples with RIN (RNA integrity) greater than 7 were used for sequencing. Paired-ended sequencing was performed on Illumina’s NovaSeq 6000 Sequencing System. HISAT2 (http://daehwankimlab.github.io/hisat2/) was used to map reads to the mouse genome, and StringTie (https://ccb.jhu.edu/software/stringtie/) was used to assemble the mapped reads. EdgeR (https://web.stanford.edu/class/bios221/labs/rnaseq/lab_4_rnaseq.html#:~:text=edgeR%20is%20concerned%20with%20differential,with%20estimating%20absolute%20expression%20levels) was used to estimate the expression levels of all transcripts. DEGs were selected with log_2_ (fold change) higher than 1 or lower than –1 and statistical significance (*P* < 0.05). DEGs were subjected for the GO and KEGG pathway enrichment analysis in R (version 4.1.1). Mouse RNA-Seq data were deposited in the NCBI’s Gene Expression Omnibus database (GEO GSE192720), as were RNA-Seq data for SAH patients (GEO GSE143318). The RNA-Seq data for mild to moderate alcohol-associated steatohepatitis patients were described previously (57).

### Statistics.

Results are expressed as mean ± SEM. All statistical analyses were performed using GraphPad Prism software (version 8.0a). Appropriate comparisons, including Student’s *t* test or 1-way ANOVA followed by post hoc analysis with Tukey’s test, were used. *P* < 0.05 was considered statistically significant.

### Study approval.

Animal care and experiments were conducted under the guidelines and protocols approved by the NIAAA and Indiana University Animal Care and Use Committees. All animals were cared for in accordance with NIH guidelines. The use of liver tissues from controls and patients with SAH was approved by the IRB at the John Hopkins University. The use of paraffin-embedded healthy control liver tissues and alcoholic cirrhosis from the Liver Tissue Cell Distribution System was approved by the IRB at the University of Minnesota. The use of paraffin-embedded liver tissues was approved by the IRB at Indiana University–Purdue University Indianapolis (protocol 1712511018).

## Author contributions

JM designed and conducted the experiments and wrote the paper. AG, BM, SH, OP, BJP, ARA, LM, PK, NH, YH, YG, and DF conducted some experiments and edited the manuscript. ZY, RS, PSB, MZ, AMC, RB, FT, and ZS helped with data analysis and edited the manuscript. BG and SL supervised the whole project and wrote the paper.

## Supplementary Material

Supplemental data

## Version 1 07/15/2022

Electronic publication

## References

[1] Singal AK (2021). Alcohol-associated liver disease in the United States is associated with severe forms of disease among young, females and Hispanics. Aliment Pharmacol Ther.

[2] Singal AK, Mathurin P (2021). Diagnosis and treatment of alcohol-associated liver disease: a review. JAMA.

[3] Szabo G (2019). Alcohol-related liver disease: areas of consensus, unmet needs and opportunities for further study. Hepatology.

[4] Crabb DW (2020). Diagnosis and treatment of alcohol-associated liver diseases: 2019 practice guidance from the American Association for the Study of Liver Diseases. Hepatology.

[5] Gao B, Bataller R (2011). Alcoholic liver disease: pathogenesis and new therapeutic targets. Gastroenterology.

[6] Mandrekar P (2016). Alcoholic hepatitis: translational approaches to develop targeted therapies. Hepatology.

[7] Gougol A (2021). Alcoholic hepatitis. Clin Liver Dis (Hoboken).

[8] Avila MA (2020). Recent advances in alcohol-related liver disease (ALD): summary of a Gut round table meeting. Gut.

[9] Mitchell MC (2020). Current management and future treatment of alcoholic hepatitis. Gastroenterol Hepatol (N Y).

[10] Sehrawat TS (2020). The knowns and unknowns of treatment for alcoholic hepatitis. Lancet Gastroenterol Hepatol.

[11] Singal AK, Shah VH (2019). Current trials and novel therapeutic targets for alcoholic hepatitis. J Hepatol.

[12] Gao B (2019). Inflammatory pathways in alcoholic steatohepatitis. J Hepatol.

[13] Abu Omar Y (2019). Prognostic value of neutrophil-lymphocyte ratio in patients with severe alcoholic hepatitis. Cureus.

[14] Xu R (2014). The role of neutrophils in the development of liver diseases. Cell Mol Immunol.

[15] Fiuza C (2000). In vivo neutrophil dysfunction in cirrhotic patients with advanced liver disease. J Infect Dis.

[16] Nemeth T (2020). Neutrophils as emerging therapeutic targets. Nat Rev Drug Discov.

[17] Cho Y, Szabo G (2021). Two faces of neutrophils in liver disease development and progression. Hepatology.

[18] Liu K (2021). Neutrophils in liver diseases: pathogenesis and therapeutic targets. Cell Mol Immunol.

[19] Chang B (2015). Short- or long-term high-fat diet feeding plus acute ethanol binge synergistically induce acute liver injury in mice: an important role for CXCL1. Hepatology.

[20] Das S (2017). Hyperoxidized albumin modulates neutrophils to induce oxidative stress and inflammation in severe alcoholic hepatitis. Hepatology.

[21] Li M (2017). MicroRNA-223 ameliorates alcoholic liver injury by inhibiting the IL-6-p47(phox)-oxidative stress pathway in neutrophils. Gut.

[22] Bukong TN (2018). Abnormal neutrophil traps and impaired efferocytosis contribute to liver injury and sepsis severity after binge alcohol use. J Hepatol.

[23] Quinn MT, Gauss KA (2004). Structure and regulation of the neutrophil respiratory burst oxidase: comparison with nonphagocyte oxidases. J Leukoc Biol.

[24] El-Benna J (2009). p47phox, the phagocyte NADPH oxidase/NOX2 organizer: structure, phosphorylation and implication in diseases. Exp Mol Med.

[25] Bertola A (2013). Chronic plus binge ethanol feeding synergistically induces neutrophil infiltration and liver injury in mice: a critical role for E-selectin. Hepatology.

[26] You M (2004). The role of AMP-activated protein kinase in the action of ethanol in the liver. Gastroenterology.

[27] Ajmo JM (2008). Resveratrol alleviates alcoholic fatty liver in mice. Am J Physiol Gastrointest Liver Physiol.

[28] Wang X (2021). MicroRNAs as regulators, biomarkers and therapeutic targets in liver diseases. Gut.

[29] He Y (2021). Neutrophil-to-hepatocyte communication via LDLR-dependent miR-223-enriched extracellular vesicle transfer ameliorates nonalcoholic steatohepatitis. J Clin Invest.

[30] Calvente CJ (2019). Neutrophils contribute to spontaneous resolution of liver inflammation and fibrosis via microRNA-223. J Clin Invest.

[31] Zhao J (2017). A missense variant in NCF1 is associated with susceptibility to multiple autoimmune diseases. Nat Genet.

[32] Dominguez M (2009). Hepatic expression of CXC chemokines predicts portal hypertension and survival in patients with alcoholic hepatitis. Gastroenterology.

[33] Liu M (2021). Super enhancer regulation of cytokine-induced chemokine production in alcoholic hepatitis. Nat Commun.

[34] Bertola A (2013). Mouse model of chronic and binge ethanol feeding (the NIAAA model). Nat Protoc.

[35] Canto C (2009). AMPK regulates energy expenditure by modulating NAD+ metabolism and SIRT1 activity. Nature.

[36] Hou X (2008). SIRT1 regulates hepatocyte lipid metabolism through activating AMP-activated protein kinase. J Biol Chem.

[37] Samala N (2019). Clinical characteristics and outcomes of mild to moderate alcoholic hepatitis. GastroHep.

[38] Peeraphatdit TB (2020). Alcohol rehabilitation within 30 days of hospital discharge is associated with reduced readmission, relapse, and death in patients with alcoholic hepatitis. Clin Gastroenterol Hepatol.

[39] Mathurin P (2021). Early liver transplantation for acute alcoholic hepatitis: We can’t say no. J Hepatol.

[40] Potts JR (2018). In vivo imaging of hepatic neutrophil migration in severe alcoholic hepatitis with (111)In-radiolabelled leucocytes. Biosci Rep.

[41] Mathurin P (1996). Survival and prognostic factors in patients with severe alcoholic hepatitis treated with prednisolone. Gastroenterology.

[42] Altamirano J (2014). A histologic scoring system for prognosis of patients with alcoholic hepatitis. Gastroenterology.

[43] Hwang S (2020). Interleukin-22 ameliorates neutrophil-driven nonalcoholic steatohepatitis through multiple targets. Hepatology.

[44] Sozio MS (2010). The role of lipid metabolism in the pathogenesis of alcoholic and nonalcoholic hepatic steatosis. Semin Liver Dis.

[45] Lin CH (2018). Oxidative stress induces imbalance of adipogenic/osteoblastic lineage commitment in mesenchymal stem cells through decreasing SIRT1 functions. J Cell Mol Med.

[46] Hu Y (2015). Protective efficacy of carnosic acid against hydrogen peroxide induced oxidative injury in HepG2 cells through the SIRT1 pathway. Can J Physiol Pharmacol.

[47] Ruderman NB (2010). AMPK and SIRT1: a long-standing partnership?. Am J Physiol Endocrinol Metab.

[48] Wang X (2021). MicroRNA-223 restricts liver fibrosis by inhibiting the TAZ-IHH-GLI2 and PDGF signaling pathways via the crosstalk of multiple liver cell types. Int J Biol Sci.

[49] Hou X (2021). Myeloid-cell-specific IL-6 signaling promotes microRNA-223-enriched exosome production to attenuate NAFLD-associated fibrosis. Hepatology.

[50] Xu MJ (2015). Fat-specific protein 27/CIDEC promotes development of alcoholic steatohepatitis in mice and humans. Gastroenterology.

[51] Guillot A (2021). Bile acid-activated macrophages promote biliary epithelial cell proliferation through integrin alpha v beta 6 upregulation following liver injury. J Clin Invest.

[52] Guillot A (2020). Deciphering the immune microenvironment on a single archival formalin-fixed paraffin-embedded tissue section by an immediately implementable multiplex fluorescence immunostaining protocol. Cancers (Basel).

[53] Schindelin J (2012). Fiji: an open-source platform for biological-image analysis. Nat Methods.

[54] Berg S (2019). ilastik: interactive machine learning for (bio)image analysis. Nat Methods.

[55] Wahlby C (2012). An image analysis toolbox for high-throughput C. elegans assays. Nat Methods.

[56] Seo W (2019). ALDH2 deficiency promotes alcohol-associated liver cancer by activating oncogenic pathways via oxidized DNA-enriched extracellular vesicles. J Hepatol.

[57] Argemi J (2019). Defective HNF4alpha-dependent gene expression as a driver of hepatocellular failure in alcoholic hepatitis. Nat Commun.

