# Supplementary material for: Distinct histopathological phenotypes of severe alcoholic hepatitis suggest different mechanisms driving liver injury and failure
Source: J Clin Invest. 2022 Jul 15;132(14):e157780. doi: 10.1172/JCI157780 (PMC9282929; doi:10.1172/JCI157780)
Supplement: Supplemental data [file jci-132-157780-s160.pdf]

Distinct histopathological phenotypes of severe alcoholic hepatitis suggest different mechanisms driving liver injury and failure

Supporting Tables and Figures

Supplementary Table S1: Baseline demographic and laboratory data of SAH patients

Variables	Severe alcoholic hepatitis (N=40)
Age (Yrs)	44.4±9.9
Gender (Male)	25
Creatinine (mg/dl)	2.3±1.4
Total bilirubin (mg/dl)	24.1±12.1
AST (U/L)	110.0±97.3
ALT (U/L)	48.2±28.8
Albumin (g/dl)	3.1±0.7
Blood neu (K/cu mm)	13.8±9.4
MELD Score	33.5±6.9

Supplementary Table S2: Baseline demographic and laboratory data of SAH patients

Variables	Severe alcoholic hepatitis (F, N=15)	Severe alcoholic hepatitis (M, N=25)	
Age (Yrs)	41.5±7.5	45.7±10.9	*P*=0.19
Creatinine (mg/dl)	2.14±1.1	2.3±1.5	*P*=0.73
Total bilirubin (mg/dl)	23.12±11.8	25.3±12.3	*P*=0.58
AST (U/L)	141.6±150.1	93.4±40.3	*P*=0.14
ALT (U/L)	42.9±27.5	52.4±29.6	*P*=0.32
Albumin (g/dl)	3.6±0.6	2.7±0.5	*P*=0.009
Blood neu (K/ cu mm)	9.4±6.8	16.5±9.9	*P*=0.07
MELD Score	33.3±4.4	33.7±8.3	*P*=0.87

Supplementary Table 3: Baseline demographic and laboratory data of patients with mild to moderate alcoholic hepatitis

Variables	Controls (N=6)	Patients with alcoholic steatohepatitis (N=12)
Age	41.8±13.5	48.6±10.7
Sex	2M/4F	9M/3F
WBC	6.6±2.2	10.3±6.8
Hemoglobin (g/dl)	13.8±1.2	10.8±2.3
Hematocrit (%)	41.4±3.1	32.2±6.3
Platelets (x10^6/ml)	320.1±94.4	236±191.3
Total bilirubin (mg/dl)	0.56±0.35	10.5±10.0
AST (U/L)	31.2±8.7	137.7±118.8
ALT (U/L)	33.8±19.4	53.2±58.4
Albumin (g/dl)	4.3±0.3	2.8±0.6
INR	1.0±0.08	1.4±0.3
MELD score	N/A	19.7±6.2

Supplementary Table 4: Primer sequence for real-time PCR

Genes	Forward primer (5'-3')	Reverse primer (5'-3')
18s	ACGGAAGGGCACCACCAGGA	CACCACCACCCACGGAATCG
a-Sma	TCCTGACGCTGAAGTATCCGATA	GGTGCCAGATCTTTTCCATGTC
Col1a1	TAGGCCATTGTGTATGCAGC	ACATGTTCAGCTTTGTGGACC
Col1a2	GGTGAGCCTGGTCAAACGG	ACTGTGTCCTTTCACGCCTTT
Col3a1	TAGGACTGACCAAGGTGGCT	GGAACCTGGTTTCTTCTCACC
Col4a1	CTGGCACAAAAGGGACGAG	ACGTGGCCGAGAATTTCACC
F4/80	CTTTGGCTATGGGCTTCCAGTC	GCAAGGAGGACAGAGTTTATCGTG
Icam-1	CAATTTCTCATGCCGCACAG	AGCTGGAAGATCGAAAGTCCG
Igf1r	GTGGGGGCTCGTGTTTCTC	GTGGGGGCTCGTGTTTCTC
Il1b	TCGCTCAGGGTCACAAGAAA	CATCAGAGGCAAGGAGGAAAAC
Il6	TCCATCCAGTTGCCTTCTTG	TTCCACGATTTCCCAGAGAAC
Ly6g	TGCGTTGCTCTGGAGATAGA	CAGAGTAGTGGGGCAGATGG
Mcp1	ATTGGGATCATCTTGCTGGT	CCTGCTGTTCACAGTTGCC
Mef2c	ATCCCGATGCAGACGATTCAG	CGGTCTCTAGGAGGAGAAACA
Mip1b	GAAACAGCAGGAAGTGGGAG	CATGAAGCTCTGCGTGTCTG
Mip1α	GTGGAATCTTCCGGCTGTAG	ACCATGACACTCTGCAACCA
Mip2	CCAACCACCAGGCTACAGG	GCGTCACACTCAAGCTCTG
Mmp13	CTTTGGCTTAGAGGTGACTGG	AGGCACTCCACATCTTGGTTT
Mt1	AAGAGTGAGTTGGGACACCTT	CGAGACAATACAATGGCCTCC
Mt2	GCCTGCAAATGCAAACAATGC	AGCTGCACTTGTCGGAAGC
Nlrp3	AAGTAAGGCCGGAATTCACC	AAAATGCCTTGGGAGACTCA
p40phox(Ncf4)	ATCGTCTGGAAGCTGCTCAA	CCCATCCATCTGCTTTTCTG
p47phox (Ncf1)	TCCTCTTCAACAGCAGCGTA	CTATCTGGAGCCCCTTGACA
p67phox(Ncf2)	TCTATCAGCTGGTTCCCACG	CTATCTGGAGCCCCTTGACA
Slc1a4	GGCATCGCTGTTGCTTACTTC	CGAGGAAAGAGTCCACTGTCT
Slc1a6	AGCAGCCACGGCAATAGTC	ATGCCAAGCTGACACCAATGA
Taz	CCCCCGCTTTGGACAGAAAAT	AGGCTGGAAATGATTGTGGAG
Tgfb	CTCCCGTGGCTTCTAGTGC	GCCTTAGTTTGGACAGGATCTG
Tnf-α	AGGCTGCCCCGACTACGT	GACTTTCTCCTGGTATGAGATAGCAAA
Vcam-1	TGAACCCAAACAGAGGCAGAGT	GGTATCCCATCACTTGAGCAGG
Vimentin	TCCACTTTCCGTTCAAGGTC	AGAGAGAGGAAGCCGAAAGC

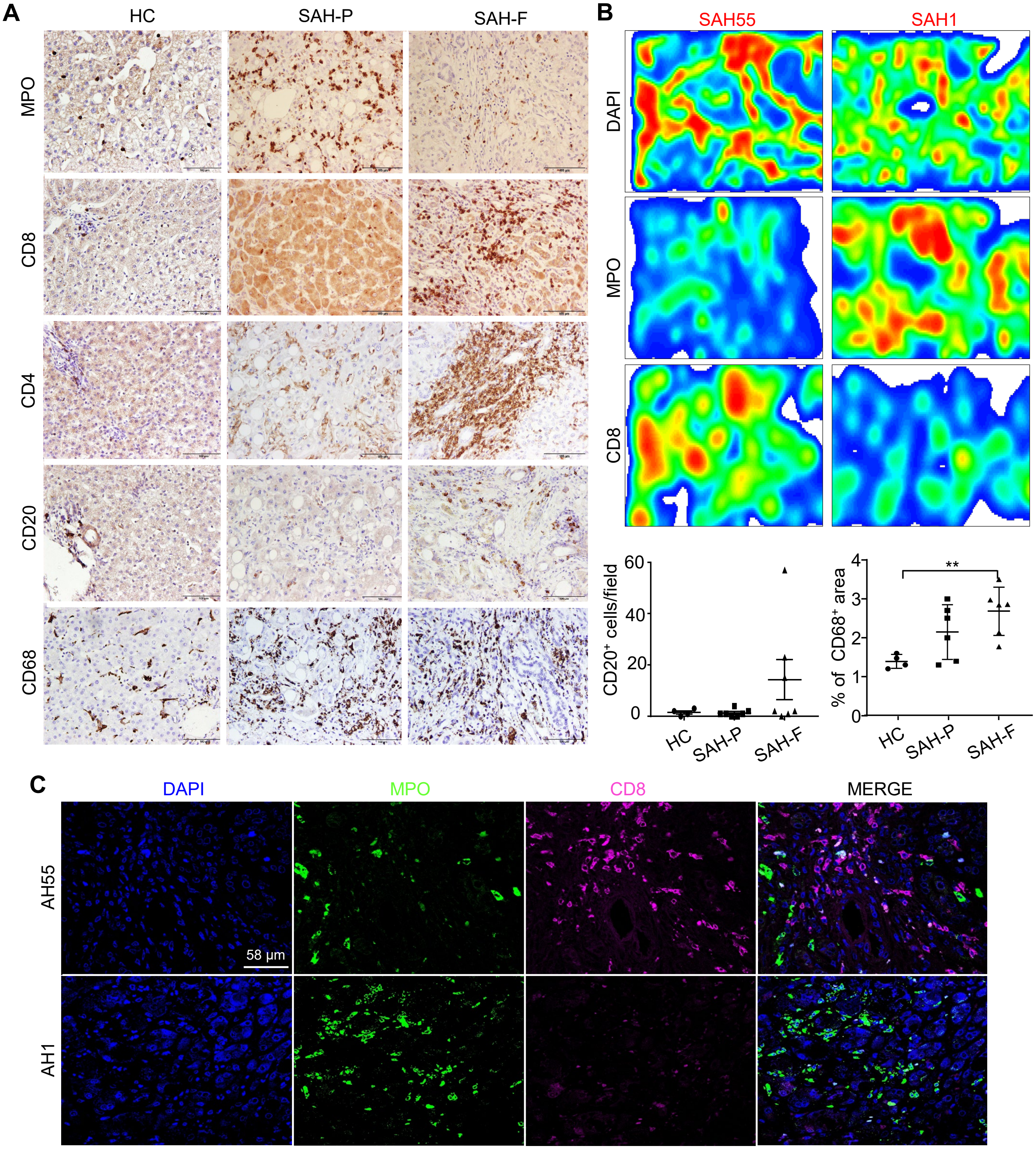

P: Parenchymal area F: Fibrotic area

Supporting Fig. S1. Immunostaining analysis of the distribution of immune cells in SAH livers. (A) Liver tissues from healthy controls (HC) (n=4) and SAH patients (n=7) were subjected to IHC staining of MPO, CD4, CD8, CD20 and C68. Representative images are shown. Scale bar: 100 µm. The number of $CD20^+$ cells and the percentage of $CD68^+$ area in parenchymal area (SAH-P) and fibrotic area (SAH-F) were quantified. **(B)** Liver tissues from SAH patients (n=10) were subjected to sequential multiplex immunofluorescence staining of HepPar1, MPO, CD8, CD4, CD20 and IBA1. Cell density map analysis of MPO and CD8 staining. **(C)** Liver tissues from SAH patients were subjected to immunofluorescence staining of MPO and CD8. Representative images are shown. Scale bar: 58 µm. Values represent the mean ± SEM. $^{**}P < 0.01$.

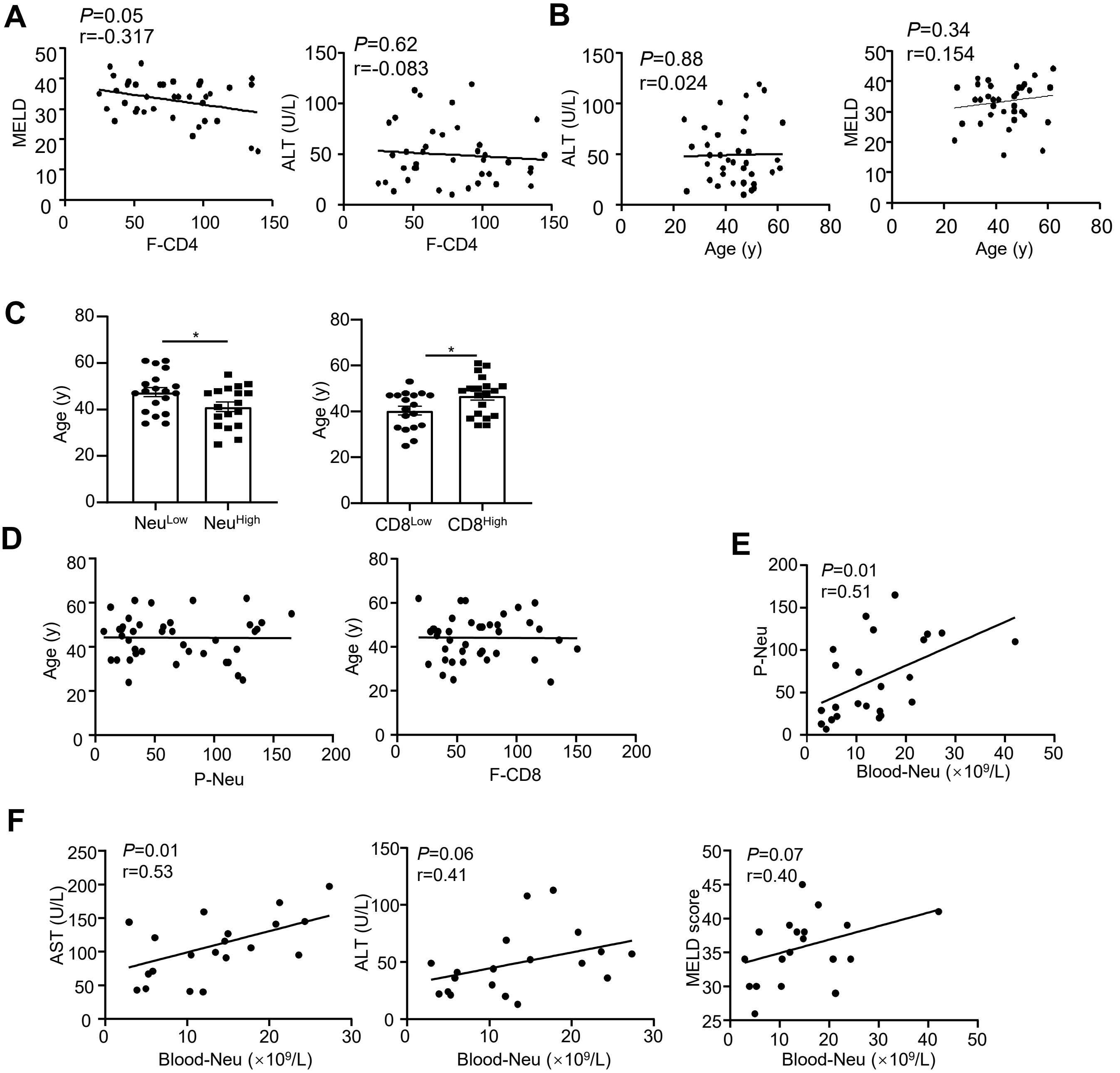

P: Parenchymal area F: Fibrotic area

Supporting Fig. S2: (A) Correlation analysis between F-CD4⁺ T cells and ALT levels or MELD score. (B) Correlation analysis between age and ALT levels or MELD score. (C) Age in SAH patients with P-Neu^{Low} and P-Neu^{High}, or F-CD8^{Low} and F-CD8^{High} group. (D) Correlation analysis between P-Neu or F-CD8⁺ T cells and age. (E) Correlation analysis between Blood-Neu and P-Neu. (F) Correlation analysis between Blood-Neu and AST, ALT levels, or MELD score. **P*<0.05

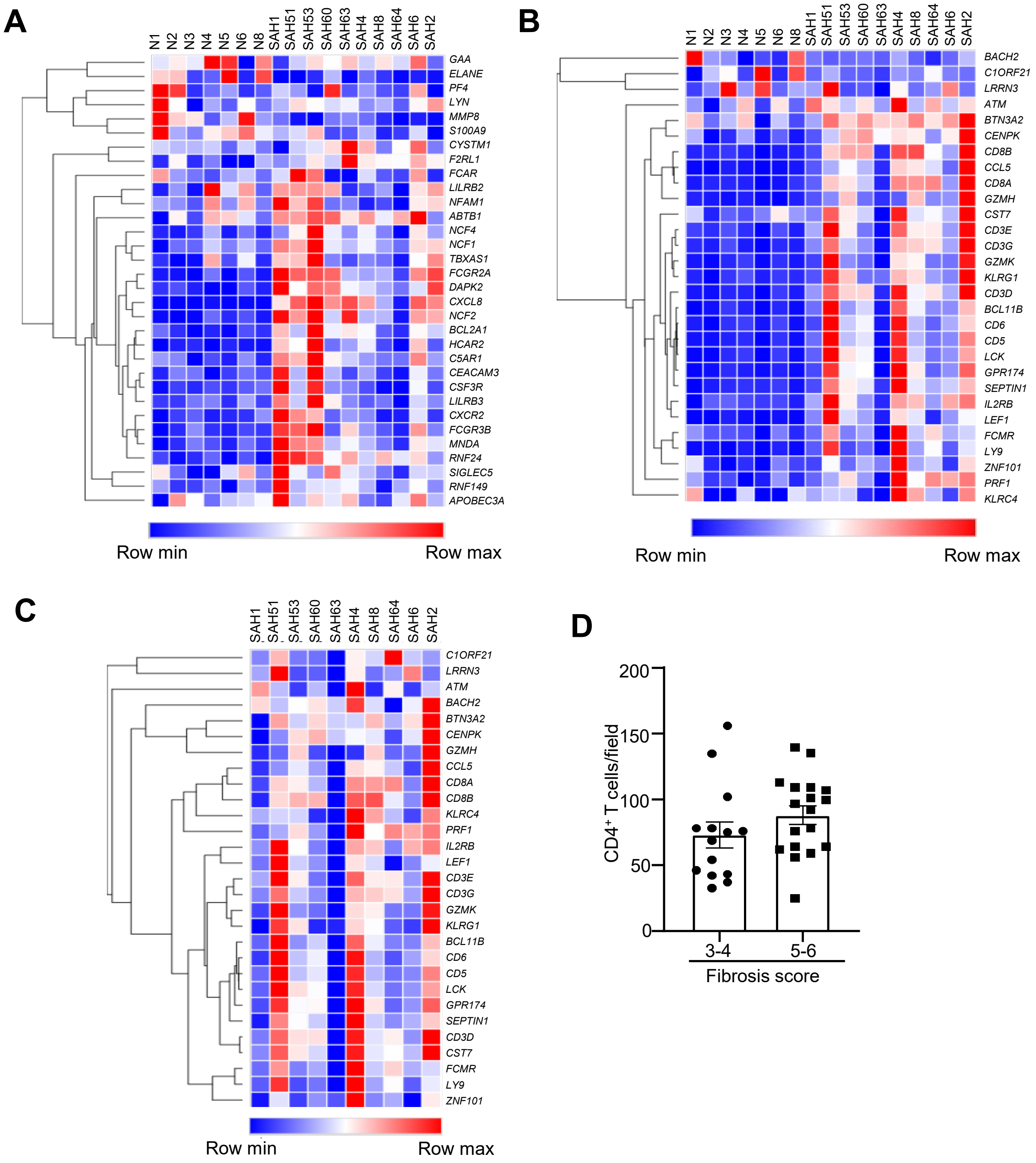

Supporting Fig. S3: Heatmap analysis of neutrophil- or CD8⁺T cell-related genes. (A, B) Heatmap analysis of neutrophil- (panel A) or CD8⁺T cell (panel B) -related genes in the liver of SAH patients and HC. **(C)** Heatmap analysis of CD8⁺ T cell-related genes in the liver among SAH patients. (D) The number of CD4⁺ T cells in the liver was compared between different fibrosis score groups.

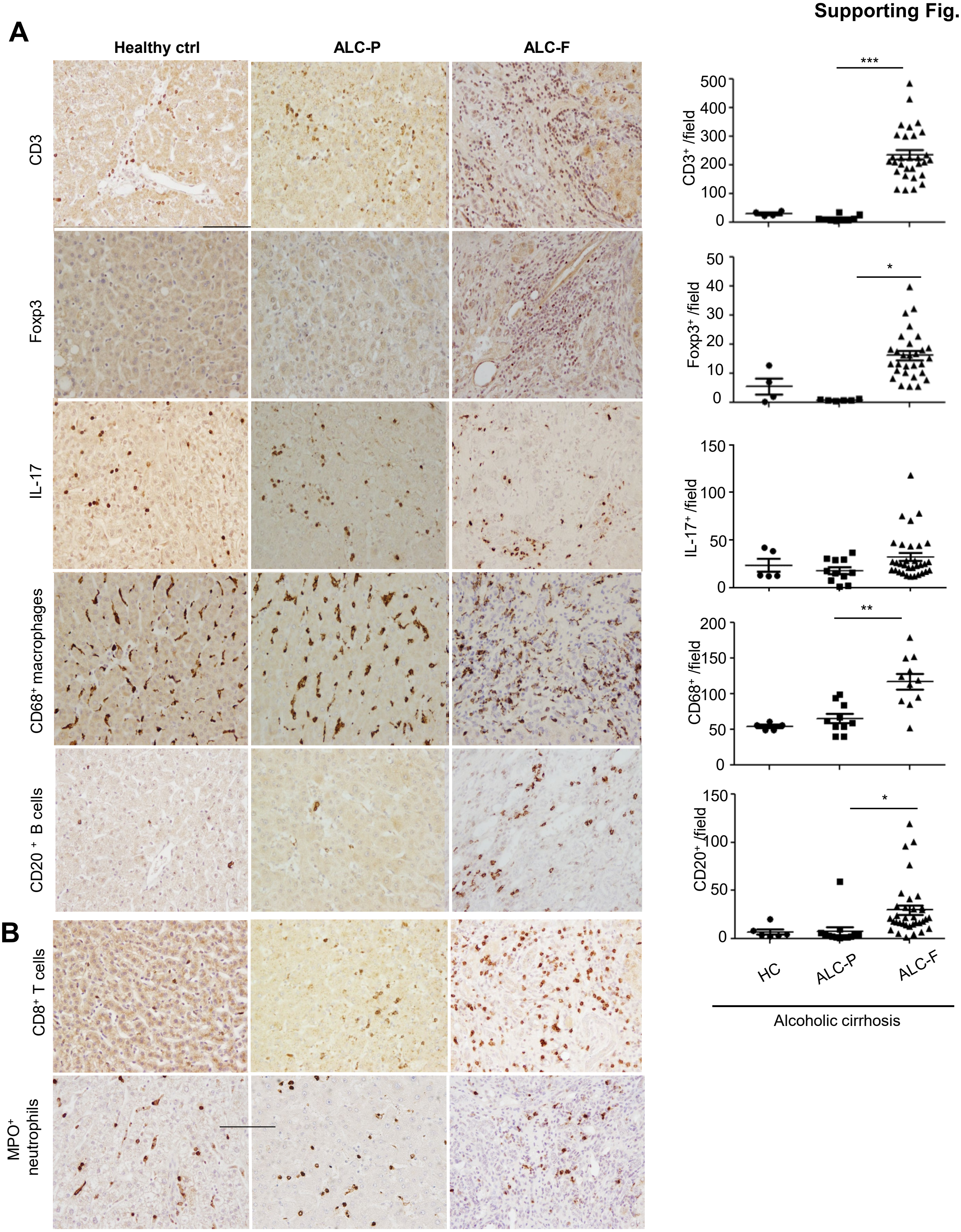

Supporting Fig. S4: Immunostaining analysis of the distribution of immune cells in the liver of ALC patients. Liver samples of healthy control (n=6) and alcoholic cirrhosis (n=32) obtained from transplanted patients were used for immunohistochemistry analysis of $CD3^+$ T cells, $Foxp3^+$ T cells, $IL17^+$ T cells, $CD68^+$ macrophages, $CD20^+$ B, $CD8^+$ and MPO^+ cells cells. Representative images are shown on the left (scale bar:100μm) and the quantification of the positive cells for each staining was shown on the right. Values represent mean ± SEM (each dot represents one sample). *P<0.05; **P<0.01; *** P<0.001. The quantification for $CD8^+$ and MPO^+ cells are shown in Fig. 1

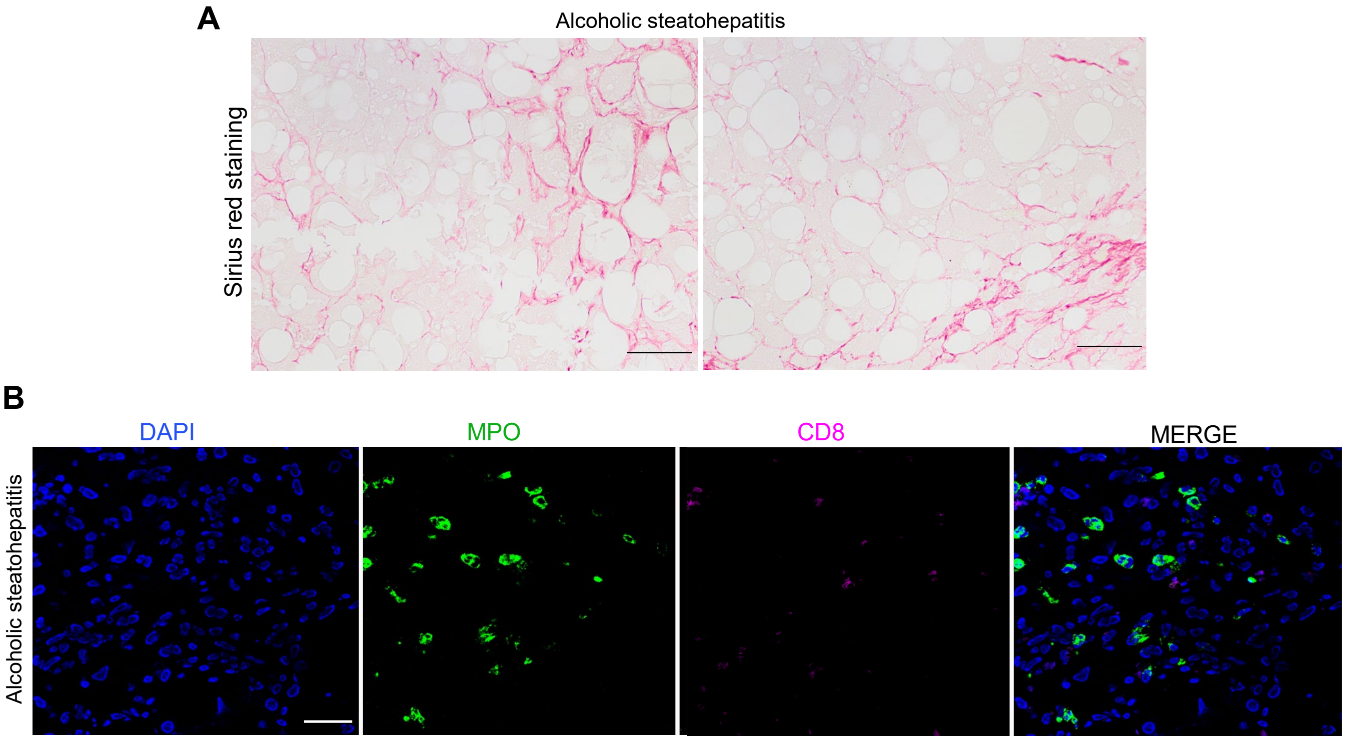

Supporting Fig. S5: Liver samples from patients with early alcoholic steatohepatitis display low levels of fibrosis and CD8^{+} T cell infiltration. (A) Liver tissues from patients with alcoholic steatohepatitis were subjected to Sirius Red staining. Representative images are shown. Scale bar: 100µm. **(B)** Liver tissues from alcoholic steatohepatitis patients were subjected to immunofluorescence staining of MPO and CD8. Representative images are shown. Scale bar: 29 µm.

A

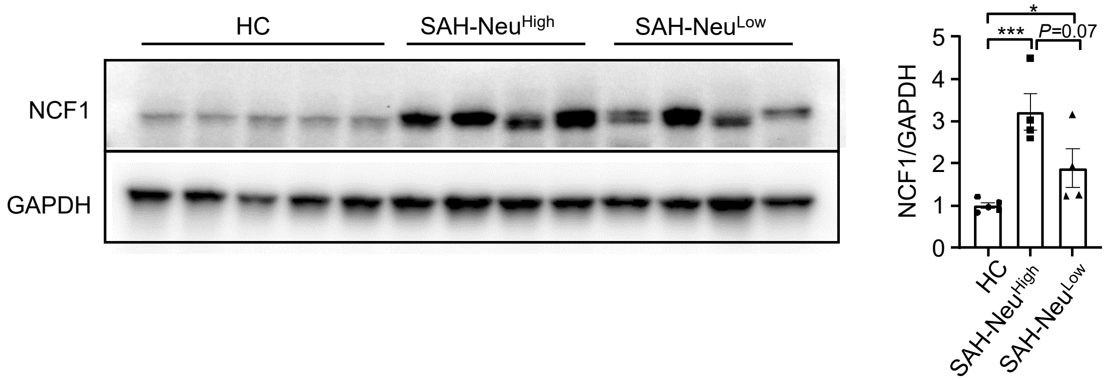

B

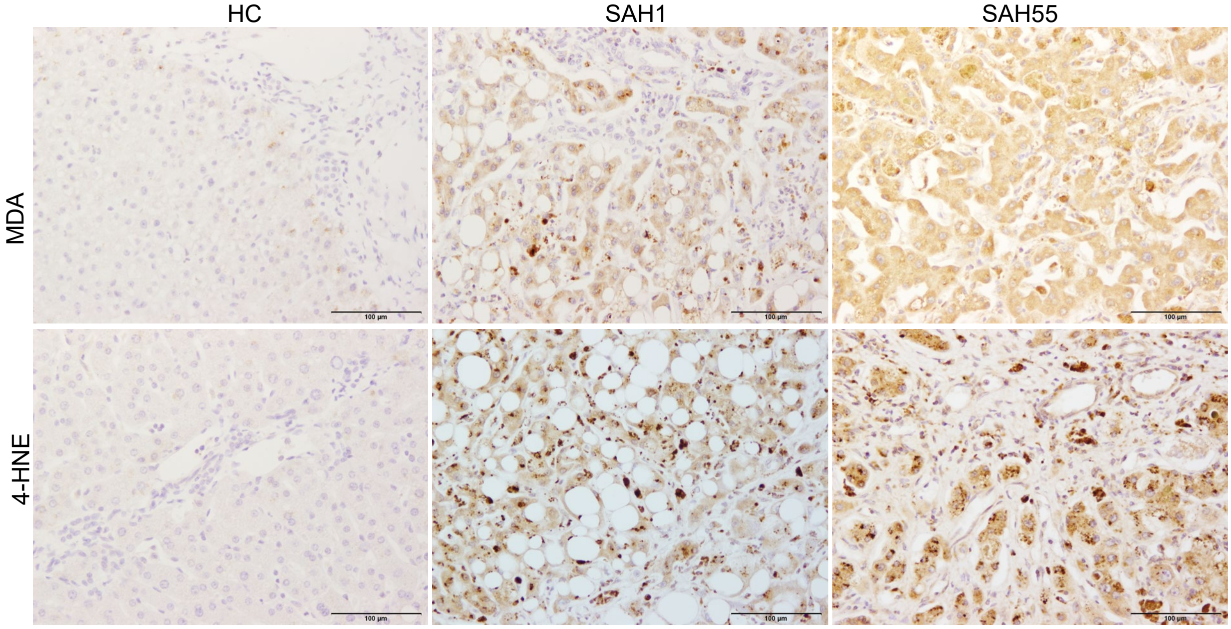

Supporting Fig. S6A-B: Hepatic levels of *p47phox/NCF1* are elevated in SAH patients and correlated with liver inflammation and oxidative stress. (A) Liver tissues from HC and SAH patients were subjected to immunoblot analysis of NCF1 and GAPDH. **(B)** Liver tissues from SAH patents were subjected to IHC analysis of MDA and HNE. Representative images are shown (scale bar:100µm).

C

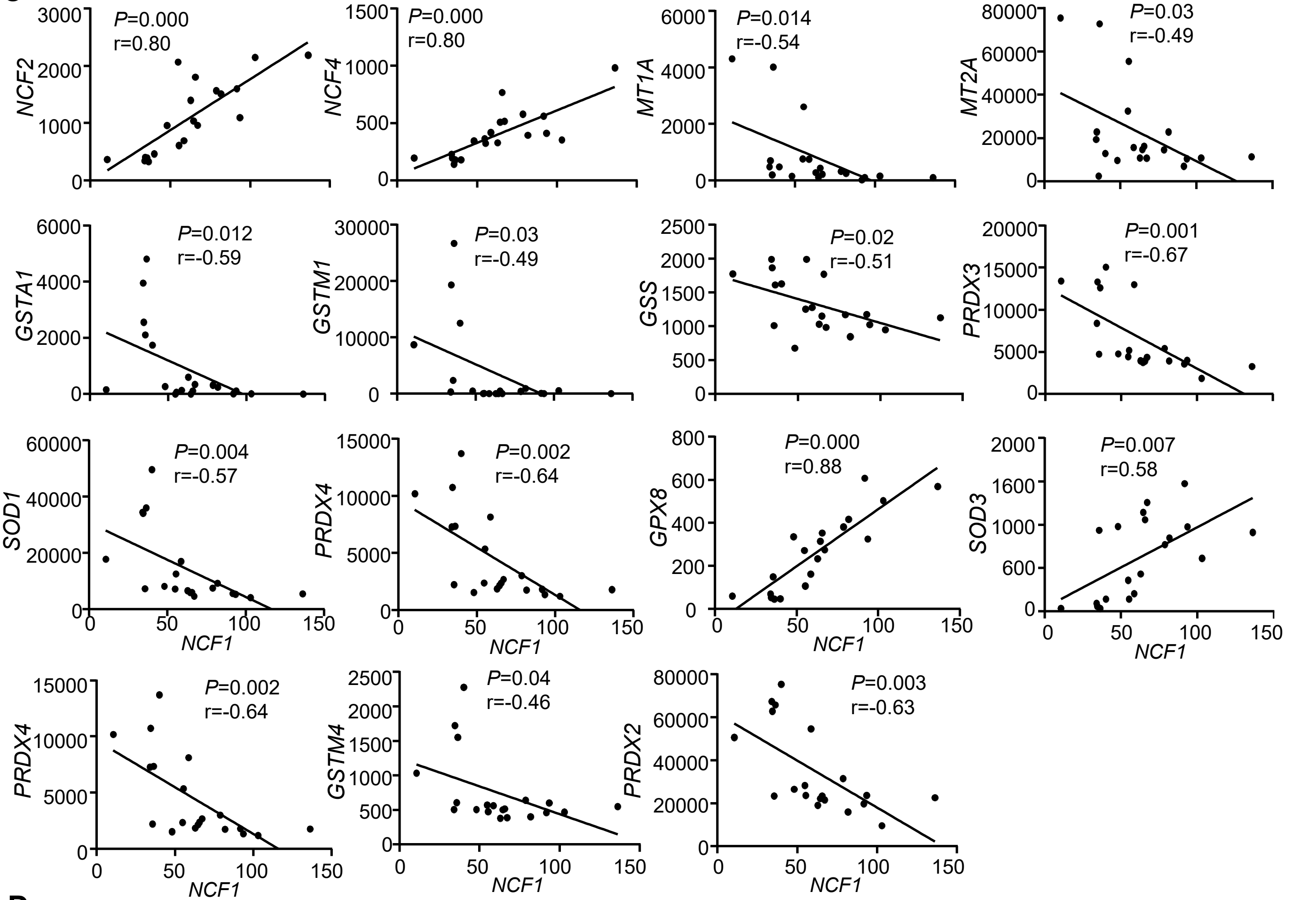

D

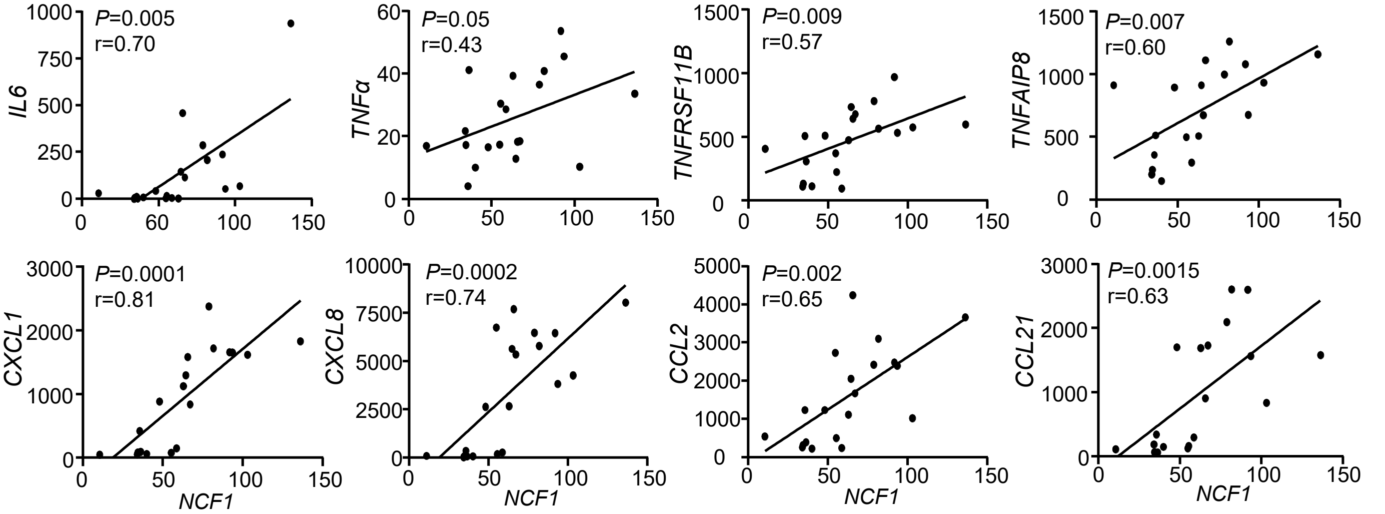

Supporting Fig. S6C-D: Hepatic levels of *p47phox/NCF1* are elevated in SAH patients and correlated with liver inflammation and oxidative stress. (C-D) Correlation analysis were performed between *NCF1* and oxidative stress related genes or inflammation related genes from SAH patients, based on RNA-seq data. The values in *X* or *Y* axis represent read counts of RNA-seq data.

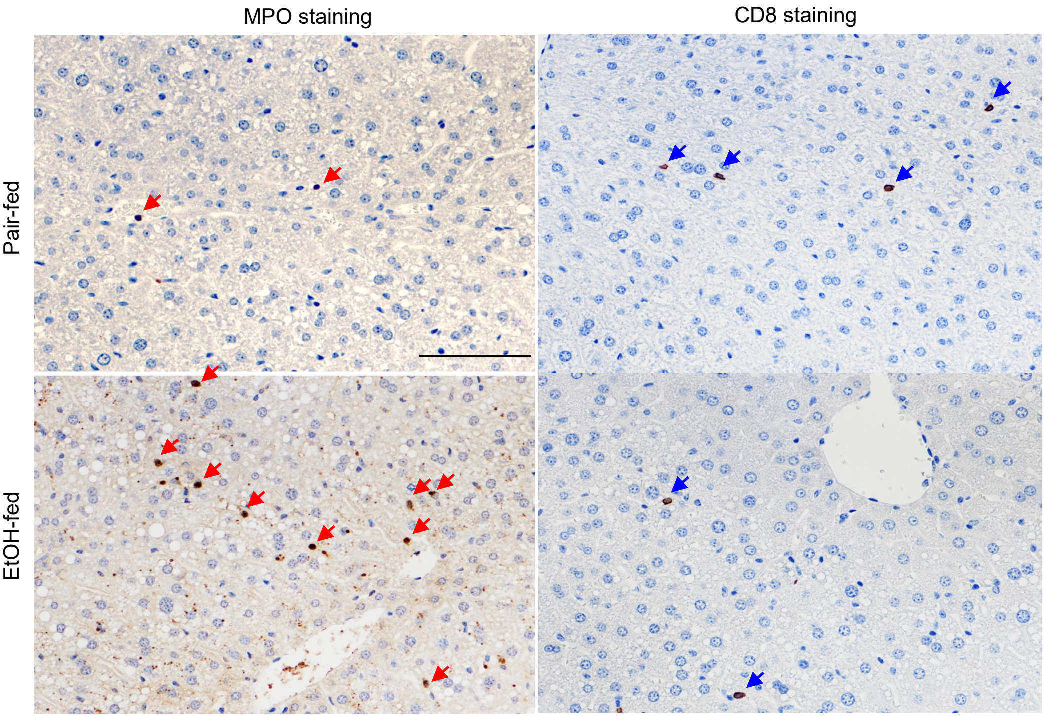

Supporting Fig. S7: C57BL6 J mice were pair-fed, ethanol-fed for E10d+1B and were euthanized 9h later. Liver tissues were subjected to immunostaining with anti-MPO and anti-CD8 antibodies. Representative images are shown (scale bar:100µm). Red arrows indicate MPO^+ neutrophils; Blue arrows indicate $CD8^+$ T cells.

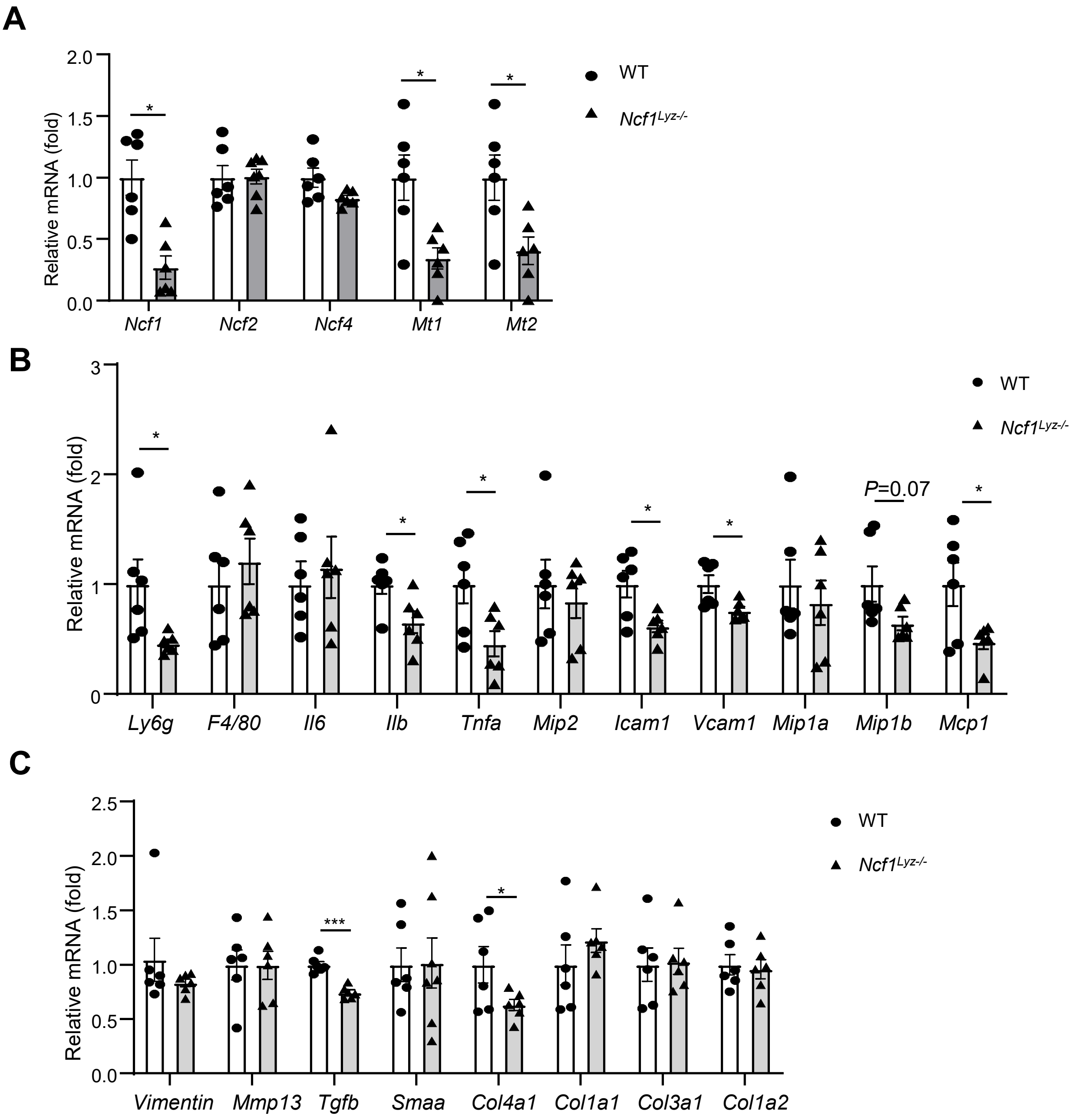

Supporting Fig. S8: Genetic deletion of the *Ncf1* gene in neutrophils ameliorates chronic-plus-binge ethanol-induced liver ROS, inflammation and fibrosis. WT and *Ncf1*$^{Lyz-/-}$ mice were fed an ethanol diet for 10 days, followed by gavage of ethanol, and were euthanized 9 hours later. RT-qPCR analysis of liver ROS related genes **(A)**, inflammation related genes **(B)**, and fibrosis related genes **(C)**. Values represent mean ± SEM. **P*<0.05, ***P*<0.01, ****P*<0.001

Supporting Fig. S9

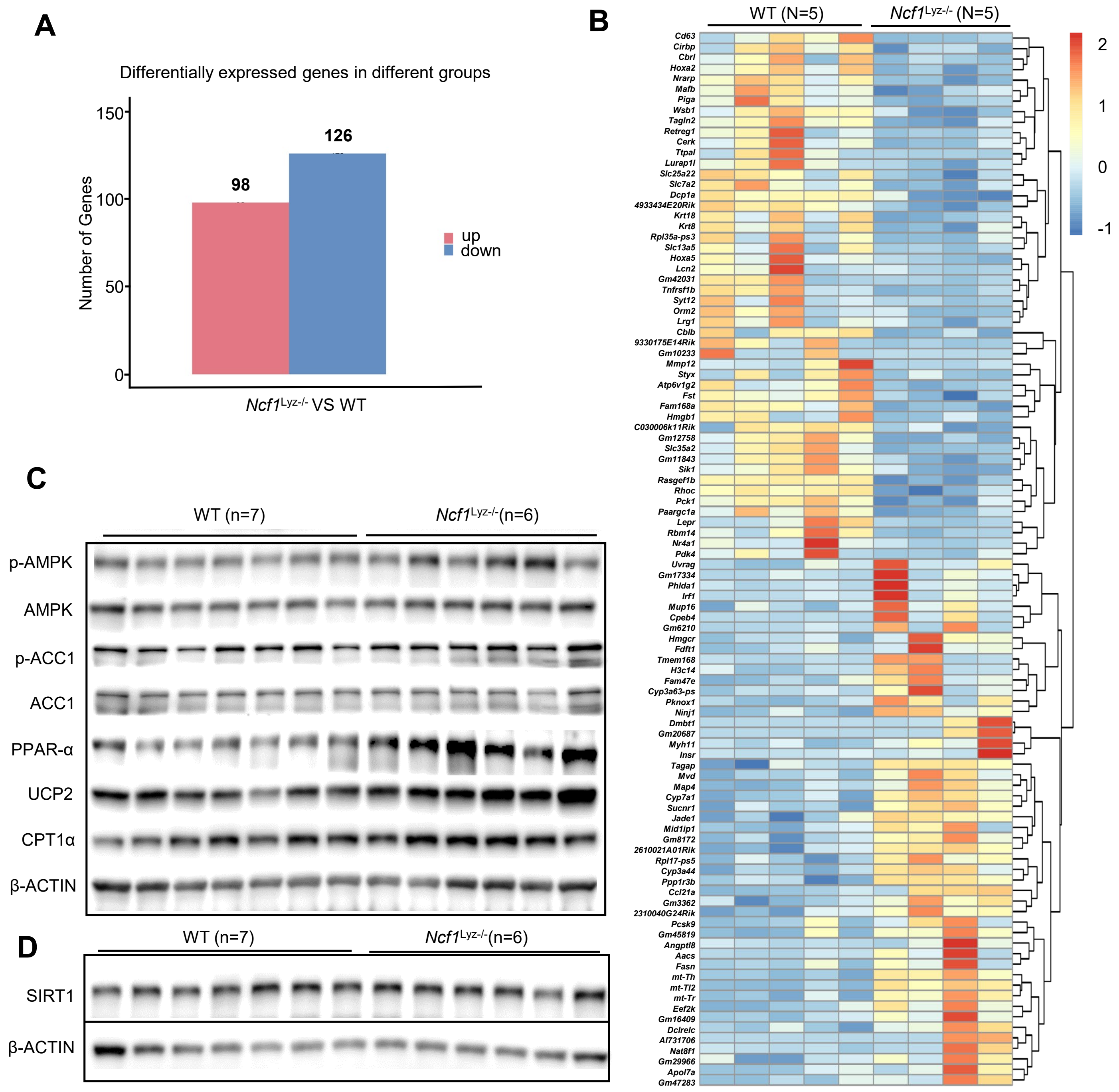

Supporting Fig. S9: A Poly(A) RNA sequencing was performed in the livers of ethanol-fed WT and *Ncf1*$^{Lyz-/-}$ mice using Illumina's NovaSeq 6000 sequencing system. The differentially expressed mRNAs (DEGs) were selected with log2 (fold change) higher than 1 or lower than -1 and statistical significance (*P* < 0.05). (A) Compared to WT mice, 98 and 126 DEGs which were up- and down-regulated in *Ncf1*$^{Lyz-/-}$ mice were identified**. (B)** Heatmap analysis of top 100 DEGs based on the *P*-value. **(C-D)** Western blot analyses of liver tissues from another independent experiment as in Fig. 8D and E.

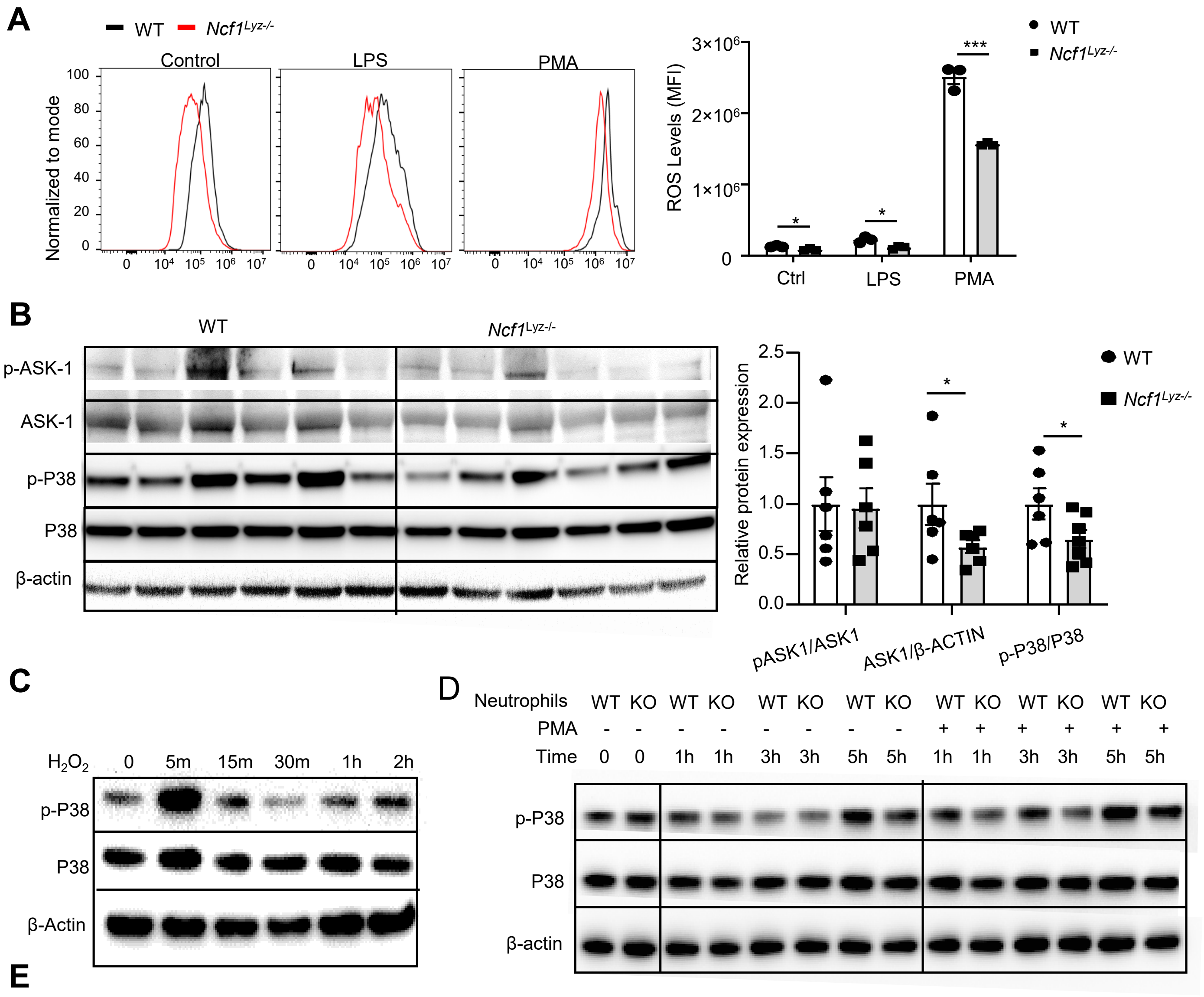

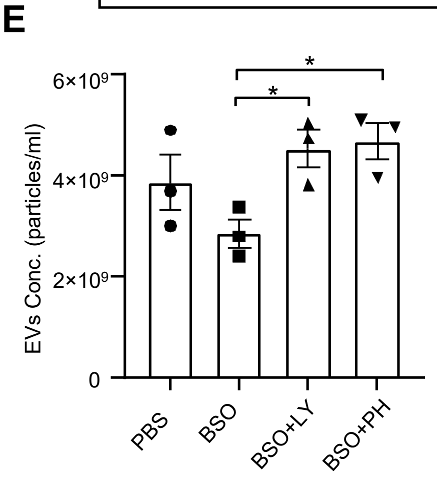

Supporting Fig. S10: Neutrophilic NCF1 regulates miR-223 expression in ROS and P38 MAPK-dependent manners. (A) Neutrophils were isolated from WT and *Ncf1*$^{Lyz-/-}$ mice and treated with LPS at 100ng/ml or PMA at 100 ng/ml for 30 minutes and ROS levels in the neutrophils were measured. **(B)** WT and *Ncf1*$^{Lyz-/-}$ mice were fed an ethanol diet for 10 days, followed by gavage of ethanol, and were euthanized 9 hours later. Western blot analysis of p-ASK1, ASK1, p-P38, P38 and β-actin in the liver. Quantification is shown on the right. **(C)** Neutrophils were isolated from WT mice and treated with H_2O_2 at 20μM. Harvested cells at 0, 5, 15, 30, 60 and 120 mins. Western blot analysis p-P38,P38 and β-actin. **(D)** Neutrophils were isolated from WT and *Ncf1*$^{Lyz-/-}$ mice and treated with or without PMA at 30ng/ml. Harvested cells at 0, 1, 3, 5 hrs. Western blot analysis p-P38,P38 and β-actin. **(E)** Neutrophils were isolated from WT mice and treated with BSO at 100 μM with or without P38α inhibitors LY2228820 (2 μM), PH797804 (4 μM) treatment. EVs concentration in the supernatant were measured. Values represent mean ± SEM. **P*<0.05 ****P* < 0.001.

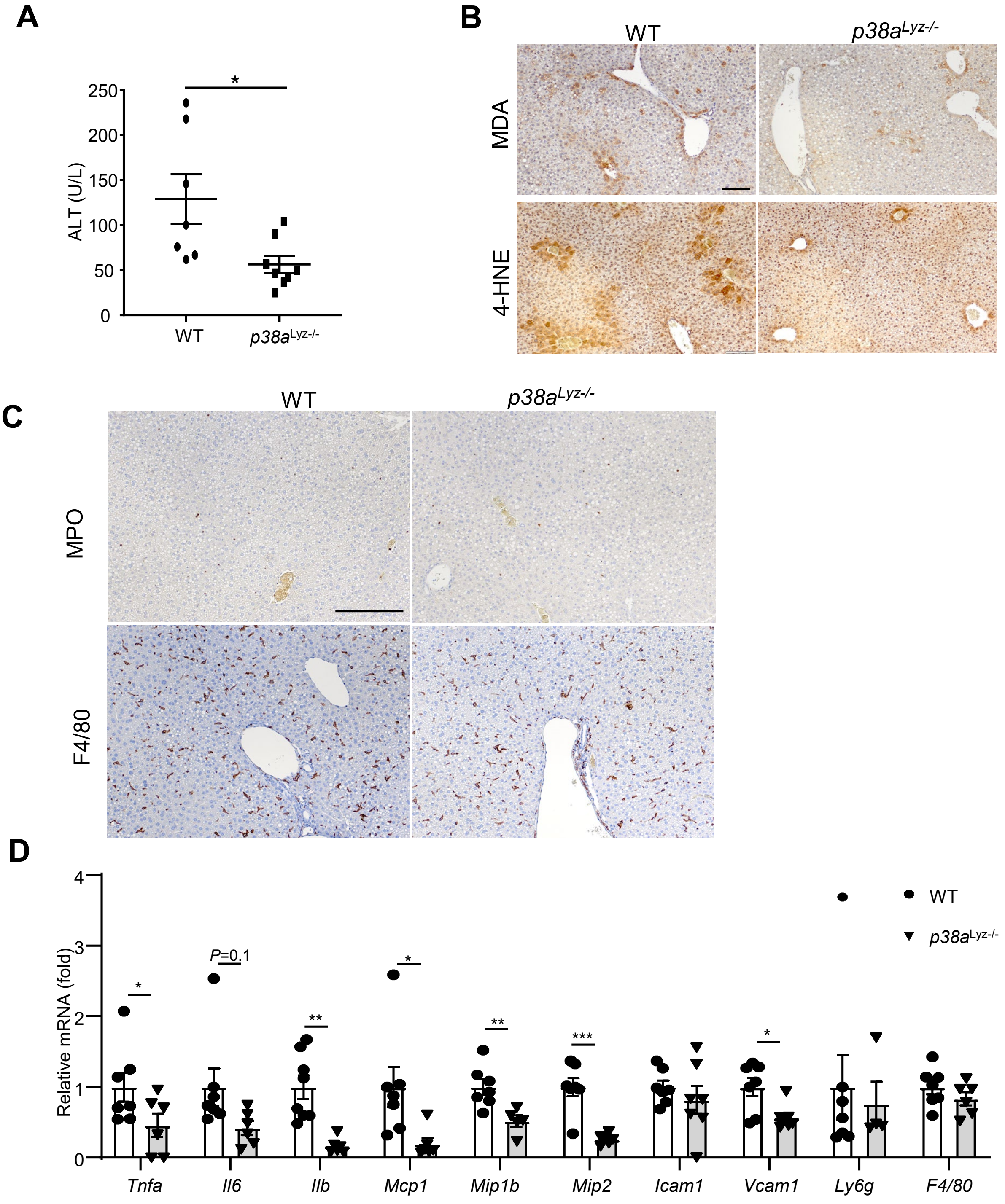

Supporting Fig. S11: Genetic deletion of the *p38a* gene in neutrophils ameliorates chronic-plus-binge ethanol-induced liver ROS, inflammation, and injury. WT and *p38a*$^{Lyz-/-}$ mice were fed an ethanol diet for 10 days, followed by gavage of ethanol, and were euthanized 9 hours later. **(A)** Serum ALT levels were measured. **(B)** Liver tissues were subjected to IHC with antibodies against MDA and HNE. Representative images are shown (scale bar:100μm). **(C)** Liver tissues were subjected to IHC with antibodies against MPO and F4/80. Representative images are shown (scale bar:200μm). **(D)** RT-qPCR analysis of liver inflammation related genes. Values represent mean ± SEM. *P< 0.05, **P < 0.01, ***P < 0.001.

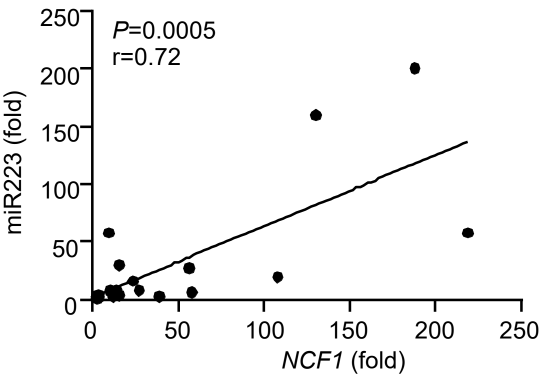

Supporting Fig. S12: Hepatic levels of *p47phox/NCF1* are positively correlated with hepatic miR223 in SAH patients. Liver tissues from SAH patients were subjected to RT-qPCR analysis of NCF1 and miR223 levels. Correlation analysis was performed between hepatic levels of NCF1 and hepatic miR223.
